# AURKA/PHB2 signaling drives acquired resistance to KRAS ^G12C^ inhibitors in KRAS ^G12C^-mutant NSCLC

**DOI:** 10.1038/s41420-026-03080-4

**Published:** 2026-04-25

**Authors:** Jinrong Liao, Xin Lan, Zeng Chen, Dan Hu, Doudou Luo, Huocong He, Zhiyi Huang, Hongyu Yu, Yunpeng Bai, Xingguang Luo, Xiandong Lin

**Affiliations:** 1 Laboratory of Radiation Oncology and Radiobiology, Clinical Oncology School of Fujian Medical University, Fujian Cancer Hospital, Fuzhou, China; 2 College of Chemistry, Fuzhou University, Fuzhou, China; 3 Department of Pathology, Clinical Oncology School of Fujian Medical University, Fujian Cancer Hospital, Fuzhou, China; 4 Key Laboratory of Microbial Pathogenesis and Interventions of Fujian Province University, the Key Laboratory of Innate Immune Biology of Fujian Province, Biomedical Research Center of South China, College of Life Sciences, Fujian Normal University, Fuzhou, China; 5 Fujian Medical University, Fuzhou, China; 6 Laboratory Animal Center. Clinical Oncology School of Fujian Medical University, Fujian Cancer Hospital, Fuzhou, China; 7 Beijing Huilongguan Hospital, Beijing University Huilongguan School of Clinical Medicine, Beijing, China; 8 NHC Key Laboratory of Cancer and Metabolism (Fujian Cancer Hospital), Fuzhou, China

**Keywords:** Non-small-cell lung cancer, Non-small-cell lung cancer

## Abstract

Patients with non-small cell lung cancer (NSCLC) who initially respond to Sotorasib, a drug targeting the KRAS ^G12C^ mutation, eventually develop acquired resistance. However, the mechanisms driving this acquired resistance remain largely unclear. This study explored the role of AURKA in mediating resistance to Sotorasib in NSCLC. The expression levels of AURKA mRNA and protein in NSCLC cell lines (H358 and Calu-1) were assessed using qPCR and Western blot. To further elucidate the role of AURKA in the biological alterations of Sotorasib-resistant cells and its association with the PI3K/AKT signaling pathway, a comprehensive set of assays was conducted, including MTS, colony formation, Transwell migration, luciferase reporter assays, fluorescent in situ hybridization (FISH), molecular docking analyses, and immunoprecipitation. The key findings include: (1) Long-term Sotorasib treatment led to upregulation of AURKA; (2) Overexpression of AURKA induced Sotorasib resistance, suppressed apoptosis and promoted migratory potential in Calu-1 and H358 cells, while AURKA knockdown increased the sensitivity, enhanced apoptosis and inhibited migratory capacity of H358-SR and Calu-1-SR cells to Sotorasib; (3) Immunoprecipitation and luciferase reporter assays demonstrated a physical interaction between AURKA and PHB2, establishing a positive feedback loop that sustained malignant behaviors, potentially explaining how Sotorasib-resistant cells survived despite KRAS pathway inhibition; (4) AURKA stabilizes PHB2, activating the PI3K/AKT pathway and allowing cancer cells to bypass the KRAS blockade, thus restoring malignant behavior. (5) The combination of AURKA inhibitor and Sotorasib alleviates the acquired drug resistance in vitro and in vivo. These data suggest that resistance to Sotorasib in NSCLC is associated with a positive feedback loop involving AURKA, PHB2, and PI3K/AKT signaling. AURKA may serve as a biomarker for predicting the therapeutic efficacy of Sotorasib in KRAS G12C-targeted therapies and as a potential therapeutic target to overcome Sotorasib resistance in NSCLC.

## Introduction

Lung cancer remains one of the most common malignancies globally, with China reporting the highest incidence, accounting for approximately 781,000 new cases annually. Non-small cell lung cancer (NSCLC) constitutes the majority of lung cancer cases, representing approximately 80–85% of all diagnoses [1, 2]. Among NSCLC patients, KRAS mutations occur with relatively high frequency, with the KRAS ^G12C^ mutation representing a distinct subtype. It is estimated that around 13% of NSCLC patients harbor KRAS ^G12C^ mutations [3], a population that typically exhibits poor response to conventional chemotherapy.

In recent years, targeted therapies have been developed specifically for KRAS ^G12C^ mutations, offering more precise treatment options. For instance, Sotorasib (AMG 510) selectively binds to KRAS ^G12C^ mutant proteins. In the CodeBreaK 100 clinical trial, Sotorasib achieved a disease control rate (DCR) of 80.6% and an objective response rate (ORR) of 37.1%. Similarly, Adagrasib (MRTX849) achieved an ORR of up to 43% and a DCR of 79% [4, 5], with enhanced brain penetration, potentially benefiting patients with brain metastases [6]. Despite these advances, drug resistance to KRAS ^G12C^ inhibitors remains a significant clinical challenge, ultimately reducing therapeutic efficacy.

The mechanisms underlying resistance to KRAS G12C inhibitors are diverse and include the following:

1. Secondary KRAS mutations: Additional mutations in the KRAS gene may alter the protein’s structure, reducing the binding affinity of targeted drugs and compromising their efficacy [7].
2. Bypass pathway activation: Alternative signaling pathways such as PI3K-AKT-mTOR or the MAPK pathway may become activated. In such cases, tumor cells continue to receive proliferative signals even when the KRAS ^G12C^ pathway is inhibited [8].
3. Alterations in the tumor microenvironment: Increased levels of cytokines like interleukin-6 (IL-6) can promote tumor survival [9], while immune cells in the microenvironment may shift from an anti-tumor to a pro-tumor phenotype, further contributing to therapy resistance [10].

Understanding these resistance mechanisms and identifying co-targeting strategies, such as combining KRAS ^G12C^ inhibitors with agents that disrupt complementary pathways, is essential for overcoming therapeutic limitations.

One promising target is Aurora kinase A (AURKA), a serine/threonine kinase encoded by a gene located at chromosomal region 20q13.2. It plays an essential role in mitotic processes such as centrosome maturation, spindle formation, and chromosome segregation [11]. Elevated AURKA expression has been observed in several solid tumors, including breast, lung, and colorectal cancers, where it is associated with enhanced tumor proliferation and poor therapeutic response [12–14]. Overexpression of AURKA can accelerate the cell cycle, reduce sensitivity to chemotherapy and radiation, and impair apoptotic responses and DNA repair mechanisms [15, 16]. In leukemia, for example, AURKA activation is linked to chemoresistance through modulation of apoptosis and DNA repair pathways [17].

In NSCLC, AURKA is implicated in mitogenic signaling and drug resistance, including reactivation of KRAS signaling and tumor cell escape from dormancy [18]. Notably, the novel Aurora A inhibitor Fangchinoline has been shown to increase cisplatin–DNA adduct formation and enhance cisplatin efficacy in ovarian cancer xenograft models [19]. Additionally, AURKA overexpression reduces sensitivity to EGFR-targeted therapies, such as gefitinib, in HCC827 NSCLC fibroblasts [20]. When EGFR signaling is suppressed by tyrosine kinase inhibitors (TKIs), AURKA may act as a compensatory mechanism, enabling continued tumor cell proliferation.

Currently, multiple AURKA inhibitors are under development, with some demonstrating therapeutic potential in hematologic malignancies and solid tumors. For example, alisertib, a selective AURKA inhibitor, has demonstrated encouraging outcomes in early clinical trials [21].

In this study, we explored the role of AURKA in mediating resistance to the KRAS ^G12C^ inhibitor Sotorasib in NSCLC cells. Our findings reveal that inhibition of the KRAS signaling pathway leads to a compensatory feedback loop involving AURKA and prohibitin 2 (PHB2), which activates the PI3K/AKT pathway to sustain cell survival, thereby contributing to drug resistance. Notably, the combined administration of Sotorasib and the AURKA inhibitor alisertib significantly suppressed proliferation in resistant NSCLC cells. These findings highlight AURKA as a potential biomarker and therapeutic target to enhance the efficacy of KRAS ^G12C^-targeted therapies in NSCLC.

## Result

### Data-driven identification of Aurka as a drug-resistance gene

**Fig. 1:**
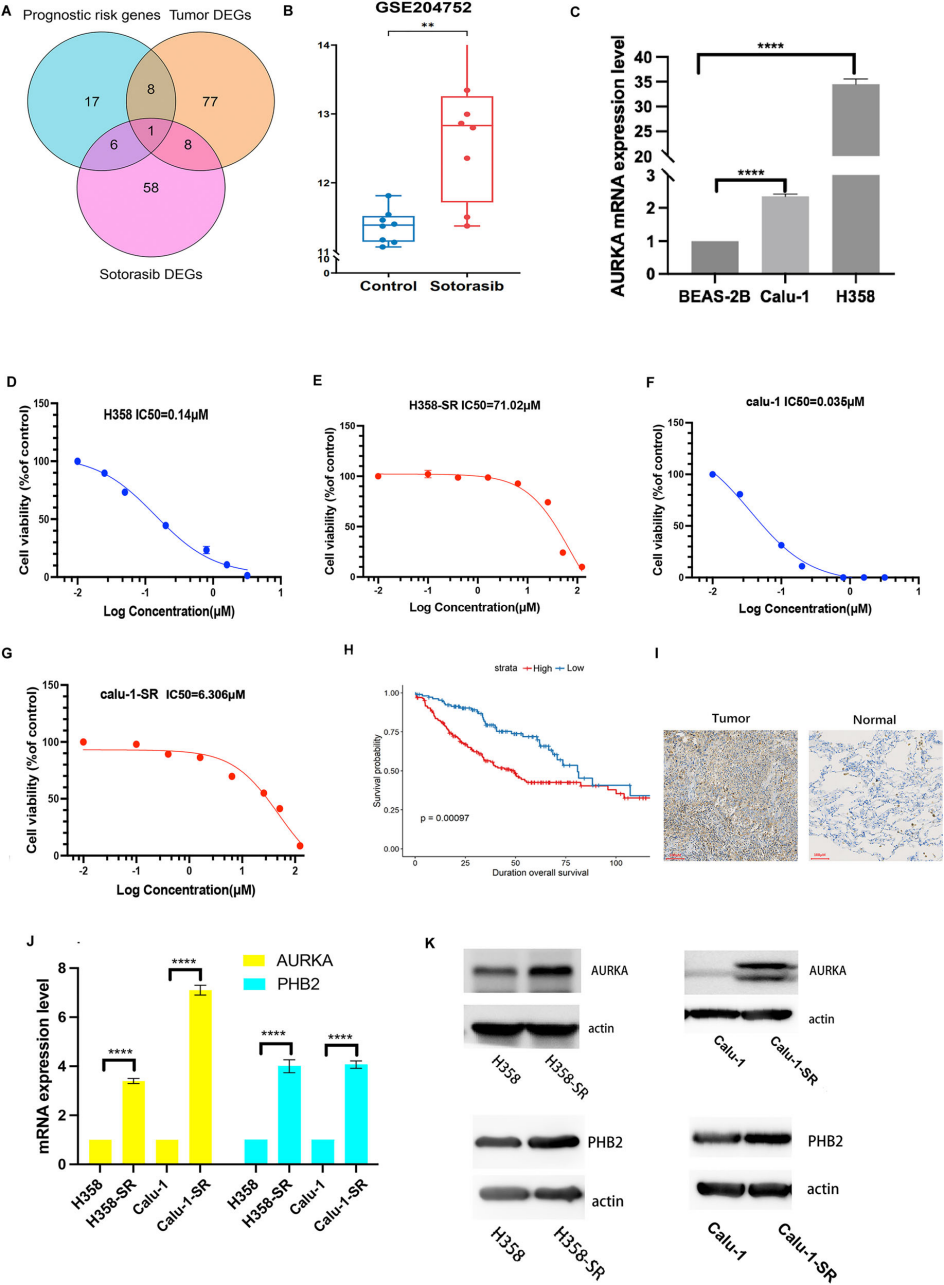
Expression of AURKA and PHB2 in Sotorasib-resistant lung cancer cell lines. **A** Intersection of prognostic risk genes, tumor-normal DEGs, and Sotorasib resistance genes revealed AURKA as the sole overlapping gene. **B** Boxplot showing AURKA expression levels in Sotorasib-resistant versus sensitive tumors from 16 Kras-G12C/Trp53 genetically engineered mice (**p < 0.01, Wilcoxon test). **C** qPCR detection of AURKA expression in normal lung epithelial cells and KRAS G12C mutant lung cancer cells H358 and Calu-1. **D – G** MTS assay for the proliferation of Sotorasib-sensitive H358 cells and Sotorasib-resistant H358-SR at various concentrations of Sotorasib, and calculation of IC50 by SPSS27. **H** Kaplan-Meier survival curves for TCGA-LUAD patients stratified by AURKA expression (high vs. low; log-rank *p* < 0.001). **I** Immunohistochemical (IHC) expression of AURKA in tumor tissues versus normal tissues from lung adenocarcinoma patients with KRAS G12C mutation. **J, K** qPCR and WB for the assessment of the expression levels of AURKA and PHB2 in H358, Calu-1, H358-SR and Calu-1-SR cells. *p < 0.05; ****p < 0.0001.

Parallel analysis of tumor versus adjacent normal tissue transcriptomes across eleven datasets identified 94 consistently dysregulated DEGs (|log2FC | ≥ 0.5, adjusted *p* < 0.01)(Table S2). Integrated analysis of single-cell and bulk RNA-seq data from Sotorasib-resistant cell lines identified 73 resistance-associated genes (Table S3). Cox proportional hazards regression analysis across six independent LUAD datasets identified genes significantly associated with patient survival. This analysis yielded signatures comprising 32 consistent risk genes (HR > 1, *p* < 0.05) across multiple cohorts(Table S4). The intersection of prognostic risk genes, tumor-normal DEGs, and resistance genes yielded AURKA as the sole overlapping gene (Fig. 1A). Transcriptomic profiling of KRAS-G12C-driven LUAD tumors from a Sotorasib-resistant mouse model revealed significant upregulation of AURKA in resistant tumors compared to controls (*p* < 0.01, Fig. 1B).

### AURKA is highly expressed in Sotorasib-resistant NSCLC cancer cell lines

The expression of AURKA was evaluated in BEAS-2B lung epithelial cells and two KRAS G12C-mutant NSCLC cell lines, Calu-1 and H358, using qRT-PCR. Both H358 and Calu-1 exhibited significantly elevated AURKA levels compared to BEAS-2B cells (Fig. 1C). To establish Sotorasib-resistant cell lines, H358 and Calu-1 cells were exposed to gradually increasing concentrations of Sotorasib over a four-month period. As a result, the IC_50_ of Sotorasib increased from 0.14 to 71.02 μM in H358 cells and from 0.035 to 6.306 μM in Calu-1 cells, representing 507.28-fold and 180.17-fold increases in resistance, respectively (Fig. 1D–G). These resistant variants were designated H358-SR and Calu-1-SR.

Kaplan-Meier survival analysis demonstrated that high AURKA expression was significantly associated with reduced overall survival (OS) across multiple datasets. In the TCGA-LUAD cohort, patients with high AURKA expression exhibited markedly reduced survival (log-rank *p* < 0.001, Fig. 1H). Consistent results were observed in GSE157009, GSE157010, GSE30219, GSE41271 and GSE42127 (Supplementary Fig. S1). We evaluated AURKA protein expression by immunohistochemistry in FFPE tumor sections and matched paired normal tissues from 25 KRAS ^G12C^-mutant lung adenocarcinoma patients. Quantitative analysis revealed a significantly elevated AURKA IHC score in tumor tissues (mean ± SD: [2.500 ± 2.623] vs [0.7037 ± 1.103]; (*p* < 0.01, by Unpaired Student’s t-test, Fig. 1I).

qRT-PCR and Western blot analysis further revealed that AURKA expression was significantly upregulated in both H358-SR and Calu-1-SR cells compared to their parental counterparts. Similarly, PHB2 expression was also significantly increased in these resistant lines (Fig. 1J, K).

In summary, both AURKA and PHB2 were upregulated in the Sotorasib-resistant H358-SR and Calu-1-SR cells.

### AURKA overexpression enhances Sotorasib resistance and promotes cell migration

**Fig. 2:**
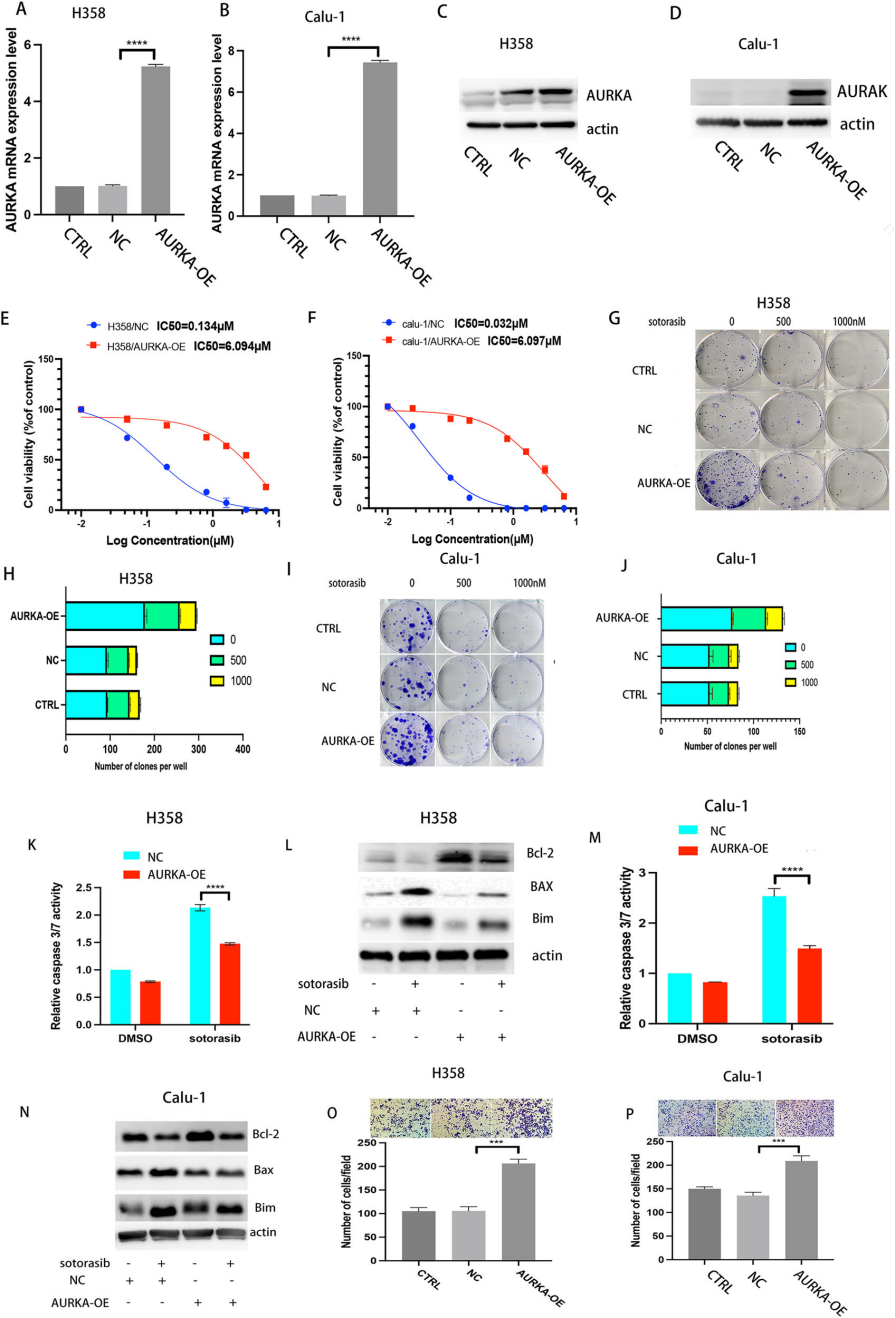
AURKA upregulation diminishes Sotorasib sensitivity in pulmonary carcinoma cells. **A**, **B** Quantitative Real-Time PCR was utilized to assess the expression levels of AURKA mRNA in H358, Calu-1, H358/NC, Calu-1/NC, H358/AURKA-OE and Calu-1/AURKA-OE. **C**, **D** Western blot analysis was employed to ascertain the expression levels of AURKA protein in H358, Calu-1, H358/NC, Calu-1/NC, H358/AURKA-OE and Calu-1/AURKA-OE. **E**, **F** The IC_50_ of H358, H358/NC, H358/AURKA-OE, Calu-1, Calu-1/NC and Calu-1/AURKA-OE cells treated with Sotorasib was measured by MTS.**G**, **J** Clonogenic assays determined the growth of Sotorasib-treated H358, H358/NC, H358/AURKA-OE, Calu-1, Calu-1/NC, and Calu-1/AURKA-OE cells. **K**, **M** Assay for Caspase 3/7 activity was employed to measure apoptosis of H358, H358/NC, H358/AURKA-OE, Calu-1, Calu-1/NC and Calu-1/AURKA-OE treated with Sotorasib. **L**, **N** Western blot analysis was used to assess the levels of apoptosis-related proteins Bcl-2, Bax, and Bim in H358, H358/NC, H358/AURKA-OE, Calu-1, Calu-1/NC, and Calu-1/AURKA-OE cells exposed to Sotorasib. **O**, **P** Transwell assays determined migration in H358, H358/NC, H358/AURKA-OE, Calu-1, Calu-1/NC, and Calu-1/AURKA-OE cell lines.

To investigate the role of AURKA in mediating Sotorasib resistance, H358 and Calu-1 cells were transduced with an AURKA-overexpressing lentivirus (AURKA-OE). qRT-PCR and Western blot confirmed successful overexpression of AURKA in H358/AURKA-OE and Calu-1/AURKA-OE cells, compared with the respective vector control groups (H358/NC and Calu-1/NC) (Fig. 2A–D).

MTS assays demonstrated that overexpression of AURKA markedly increased Sotorasib resistance. The IC50 values of Sotorasib were:

- H358/NC: 0.134 uM
- H358/AURKA-OE: 6.094uM
- Calu-1/NC:0.032 uM
- Calu-1/AURKA-OE: 6.097 nM (Fig. 2E–F)

Moreover, colony formation assays demonstrated that AURKA-overexpressing cells displayed significantly enhanced clonogenic potential in the presence of Sotorasib (Fig. 2G–J). In parallel, AURKA overexpression reduced Sotorasib-induced apoptosis, evidenced by decreased caspase 3/7 activity, increased expression of the anti-apoptotic protein Bcl-2, and decreased levels of the pro-apoptotic proteins BAX and Bim (Fig. 2K–N).

Finally, Transwell assays showed that AURKA overexpression significantly enhanced the migratory capacity of H358 and Calu-1 cells (Fig. 2O, P).

Collectively, these findings suggest that AURKA overexpression promotes resistance to Sotorasib, suppresses apoptosis, and promotes migratory potential in NSCLC cells.

### Knockdown of AURKA in Sotorasib-resistant H358-SR and Calu-1-SR cells reduces drug tolerance and inhibits cell migration

**Fig. 3:**
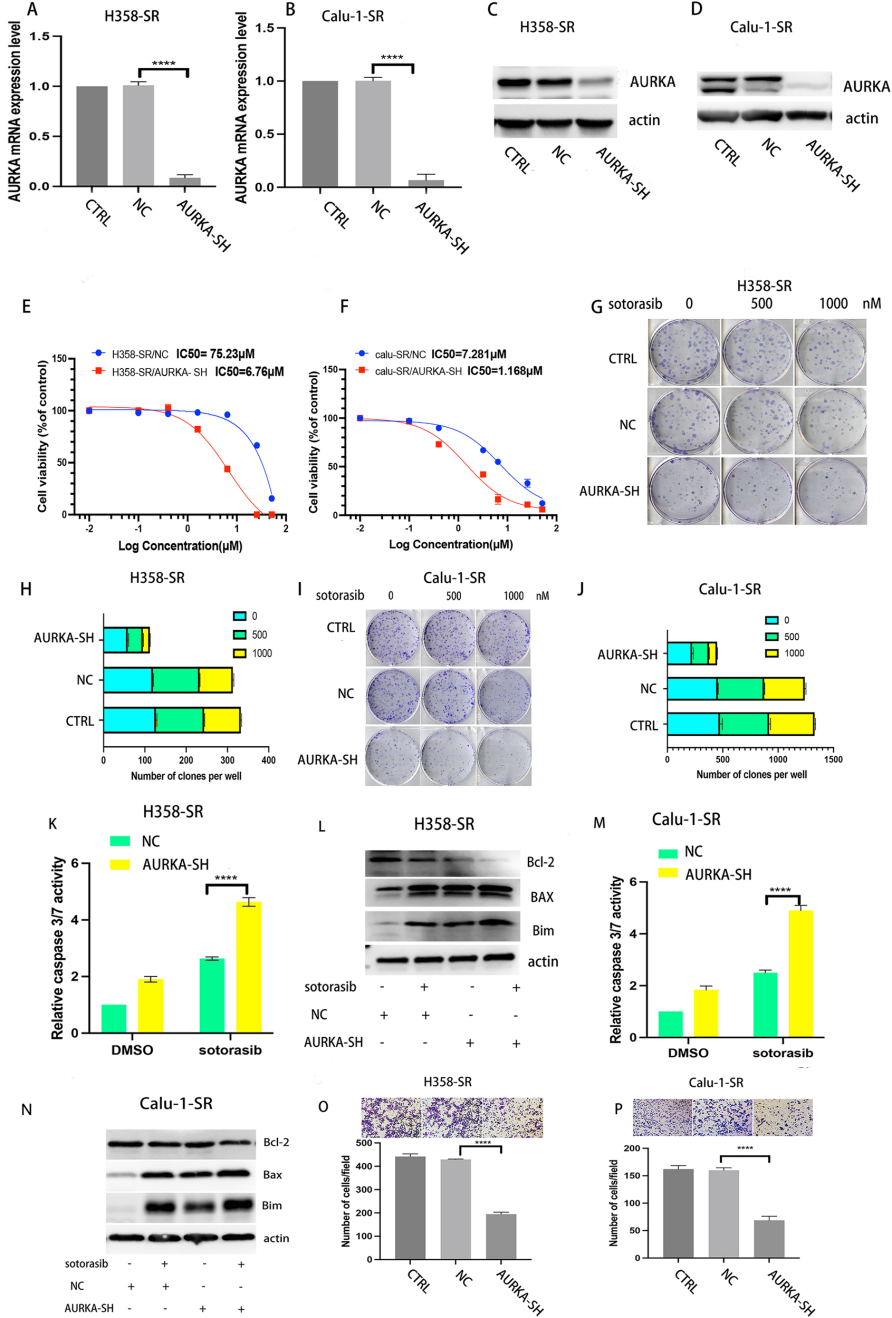
Knockdown of AURKA reduces the resistance of lung cancer cells to Sotorasib. **A**, **B** Quantitative Real-Time PCR was utilized to assess the expression levels of AURKA mRNA in H358-SR, Calu-1-SR, H358-SR/NC, Calu-1-SR/NC, H358-SR/AURKA-SH and Calu-1-SR/AURKA-SH. **C**, **D** Western blot analysis revealed the AURKA protein’s expression in H358-SR, Calu-1-SR, H358-SR/NC, Calu-1-SR/NC, H358-SR/AURKA-SH, and Calu-1-SR/AURKA-SH samples. **E**, **F** The IC_50_ of H358-SR, H358-SR/NC, H358-SR/AURKA-SH, Calu-1-SR, Calu-1-SR/NC and Calu-1-SR/AURKA-SH cells treated with Sotorasib was measured by MTS. **G–J** Colony formation assay was used to detect the cell growth ability of H358-SR, H358-SR/NC, H358-SR/AURKA-SH, Calu-1-SR, Calu-1-SR/NC and Calu-1-SR/AURKA-SH treated with Sotorasib. **K–M** Assay for Caspase 3/7 activity was employed to measure apoptosis of H358-SR, H358-SR/NC, H358-SR/AURKA-SH, Calu-1-SR, Calu-1-SR/NC and Calu-1-SR/AURKA-SH treated with Sotorasib. **L–N** Western blotting was used to determine the levels of apoptosis-related proteins – Bcl-2, Bax, and Bim – in Sotorasib-treated H358-SR, H358-SR/NC, H358-SR/AURKA-SH, Calu-1-SR, Calu-1-SR/NC, and Calu-1-SR/AURKA-SH cells. **O**, **P** Transwell assay was employed to assess the migration ability of H358-SR, H358-SR/NC, H358-SR/AURKA-SH, Calu-1-SR, Calu-1-SR/NC and Calu-1-SR/AURKA-SH cells.

To further examine the relationship between AURKA expression and Sotorasib resistance, a lentiviral shRNA construct targeting AURKA was introduced into Sotorasib-resistant H358-SR and Calu-1-SR cells. qRT-PCR and Western blot analyses confirmed that AURKA expression was significantly reduced in H358-SR/AURKA-SH and Calu-1-SR/AURKA-SH cells compared to their respective vector controls, H358-SR/NC and Calu-1-SR/NC (Fig. 3A–D).

To evaluate whether AURKA knockdown affects Sotorasib sensitivity, the IC_50_ values were determined using the MTS viability assay. AURKA knockdown significantly decreased the IC_50_ of Sotorasib:

- H358-SR/NC: 76.23uM
- H358-SR/AURKA-SH: 6.76uM
- Calu-1-SR/NC: 7.281 uM
- Calu-1-SR/AURKA-SH: 1.168uM (Fig. 3E, F)

Colony formation assays revealed that AURKA silencing markedly reduced clonogenic capacity in both H358-SR and Calu-1-SR cells treated with Sotorasib (Fig. 3G–J). Furthermore, caspase 3/7 activity was significantly elevated in AURKA-knockdown cells compared to control cells under the same treatment conditions, suggesting increased apoptosis (Fig. 3K, M). Consistently, Bim expression was upregulated, while Bcl-2 was downregulated following AURKA knockdown (Fig. 3L, N).

Finally, Transwell migration assays demonstrated a significant reduction in cell migration in AURKA-silenced H358-SR and Calu-1-SR cells, as evidenced by the decreased number of cells traversing the polyester membrane (Fig. 3O, P).

In summary, knockdown of AURKA in Sotorasib-resistant NSCLC cells increased their sensitivity to Sotorasib, enhanced apoptotic activity, and inhibited migratory capacity.

### AURKA and PHB2 mutually regulate each other through a positive feedback loop

**Fig. 4:**
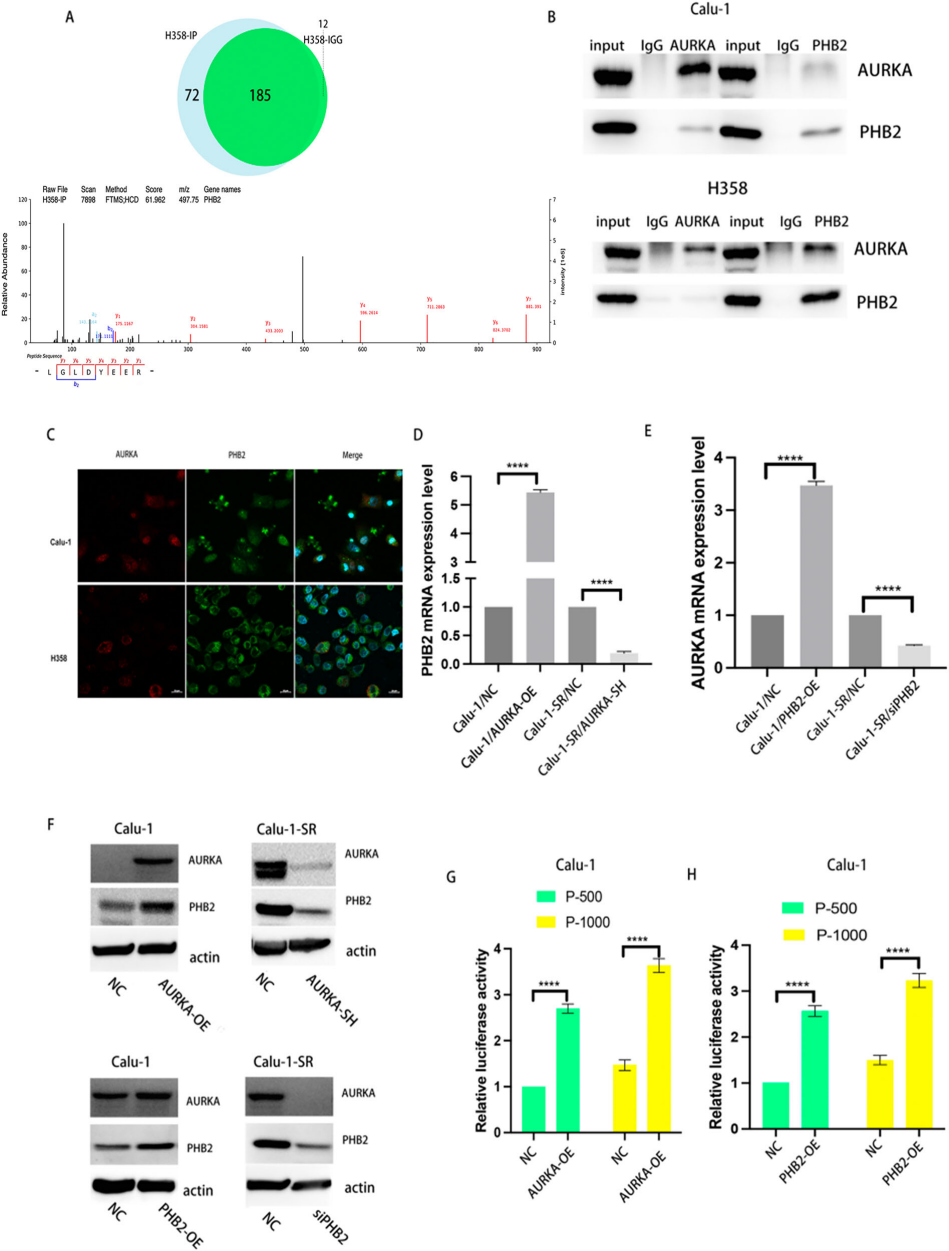
AURKA interacts with PHB2 and regulates the stability of PHB2 in Calu-1 cells. **A** Whole Calu-1 lysates were generated for immunoprecipitation with anti-AURKA antibodies or a control IgG, followed by LC-MS/MS analysis of the immunocomplexes. A total of 72 potential interacting proteins with high fidelity were identified. PHB2 emerged as a possible AURKA-interacting protein. **B** Endogenous co-immunoprecipitation assays were conducted to explore the interaction between AURKA and PHB2 in Calu - 1 cells. **C** Representative immunofluorescence images were obtained to demonstrate the colocalization of AURKA and PHB2 in H358 and Calu - 1 cells. **D**, **E** qRT-PCR was employed to detect the mRNA expression levels of PHB2 or AURKA in Calu-1 or Calu-1-SR cells. These cells had either stable knockdown or overexpression of AURKA or PHB2. **F** WB was utilized to detect the protein expression levels of PHB2 or AURKA in Calu - 1 or Calu - 1 - SR cells with stable knockdown or overexpression of AURKA or PHB2. **G**, **H** A luciferase promoter assay was performed.

To elucidate the molecular mechanism by which AURKA modulates Sotorasib resistance in NSCLC cells, we aimed to identify potential AURKA-interacting proteins. Immunoprecipitation (IP) was performed on whole-cell lysates from H358 cells using either an anti-AURKA antibody or control IgG, followed by LC-MS/MS analysis (Fig. 4A). This approach identified 72 high-confidence candidate interactors (Table S4). Among them, PHB2—a protein implicated in tumorigenesis—was selected for further investigation.

Co-IP assays confirmed the interaction between AURKA and PHB2. Using an anti-PHB2 antibody, we successfully pulled down endogenous AURKA (Fig. 4B), and the reciprocal Co-IP with anti-AURKA antibody confirmed the interaction. Immunofluorescence imaging revealed co-localization of AURKA and PHB2 in Calu-1 cells (Fig. 4C), supporting their interaction within the same cellular compartments.

To predict the mode of direct interaction between AURKA and PHB2 proteins, molecular docking analyses were conducted in this study. Experimental results demonstrated that these two proteins exhibit strong binding affinity and possess multiple specific amino acid interaction sites, providing key structural insights into their functional collaboration (Fig. S2).

Next, we examined whether AURKA and PHB2 regulate each other’s expression. Calu-1 cells with stable overexpression or knockdown of AURKA or PHB2 were analyzed via qRT-PCR and Western blot. AURKA overexpression led to a significant increase in PHB2 mRNA and protein levels, whereas AURKA knockdown markedly reduced PHB2 expression(Fig. 4D–F). Similarly, PHB2 overexpression upregulated AURKA, while its knockdown suppressed AURKA expression, suggesting mutual regulation(Fig. 4E, F).

To further validate this regulatory loop, dual-luciferase reporter assays were conducted. AURKA overexpression enhanced PHB2 promoter activity ~200-fold, and PHB2 overexpression likewise stimulated AURKA promoter activity by a similar magnitude(Fig. 4G-H).

**Fig. 5:**
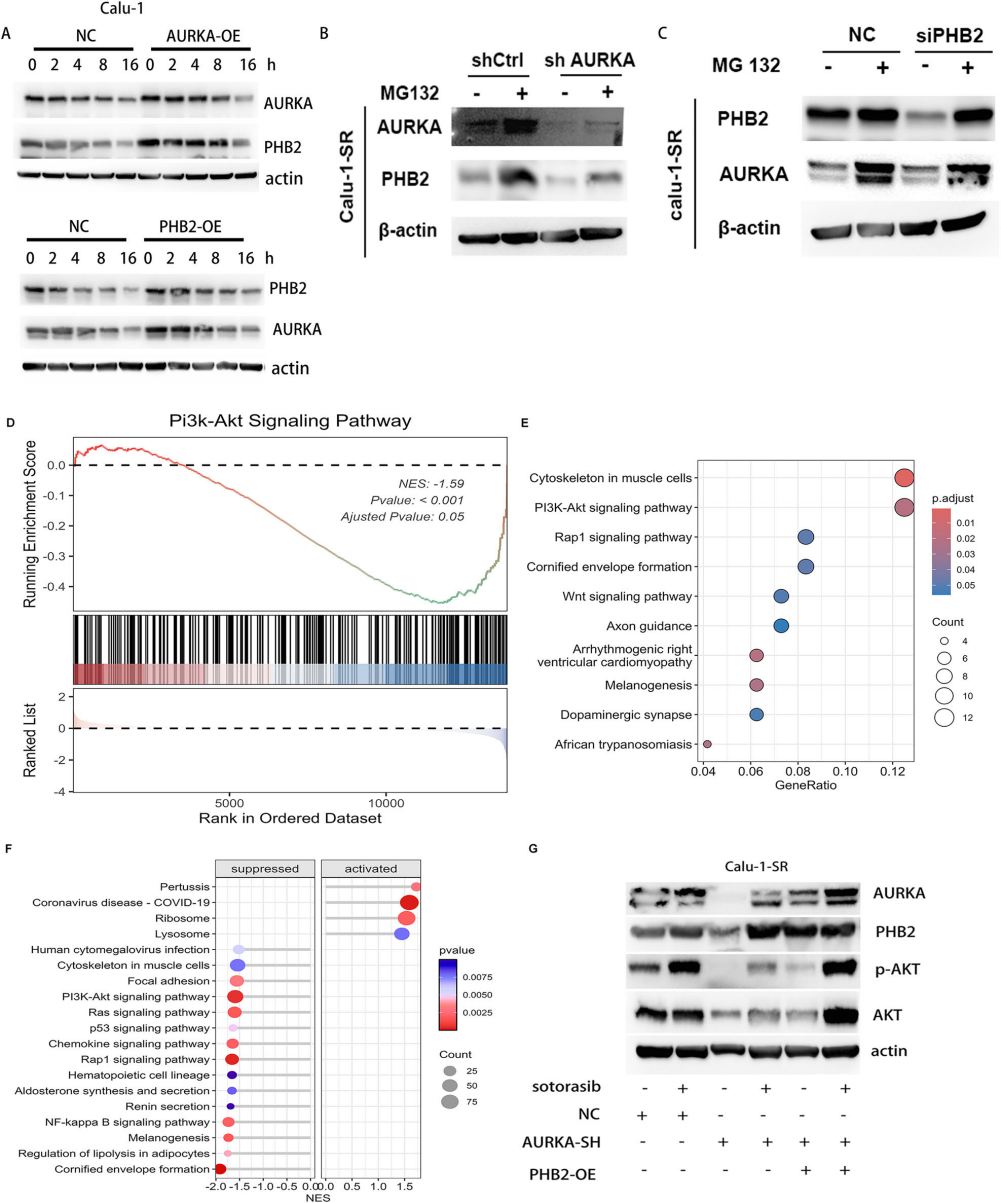
Interaction between AURKA and PHB2 and their correlation with the PI3K-Akt signaling pathway. **A** Immunoblot analysis of PHB2, AURKA, and β-actin in CHX-treated (10 µg/mL) Calu-1 cells stably expressing AURKA at specified intervals. The half - life of PHB2 was evaluated. **B, C** WB was used to detect the expression of PHB2 and AURKA in Calu - 1 cells. These cells had stable knockdown of AURKA or were treated with siPHB2. They were then treated with CHX and MG132 (a proteasome inhibitor, 10 μM) for 8 h. **D** GSEA enrichment plot of the PI3K-Akt signaling pathway in AURKA-knockdown tumor samples compared to control tumors. The pathway was significantly suppressed (NES = −1.58, *p* < 0.001, adjusted *p* < 0.05), with a distinct depletion of pathway genes at the lower end of the ranked DEG list. **E** Gene ratio plot illustrating the proportion of pathway genes in the PI3K-Akt signaling pathway relative to other enriched pathways (adjusted *p* < 0.05). **F** Over-Representation Analysis (ORA) of KEGG pathways showing significant suppression of oncogenic pathways and activation of immune-related pathways following AURKA knockdown ((adjusted *p* < 0.05). **G** Detection of the expression of AKT and p - AKT in Calu - SR cells of the NC, AURKA - SH and AURKA - SH/PHB2 - OE groups after 24-h treatment with Sotorasib by WB.

We next explored whether AURKA and PHB2 influence each other’s protein stability through post-translational mechanisms. Calu-1 cells were treated with cycloheximide (CHX), a protein synthesis inhibitor (Fig. 5A). AURKA overexpression significantly prolonged the half-life of PHB2 protein. Additionally, MG132, a proteasome inhibitor, was used to determine whether this effect involves the ubiquitin-proteasome pathway. In AURKA-knockdown cells, PHB2 protein levels were substantially decreased, but MG132 treatment restored PHB2 expression (Fig. 5B, C), suggesting AURKA helps prevent PHB2 degradation via the proteasome. The same pattern was observed in PHB2-knockdown cells, where AURKA levels dropped significantly, but were rescued by MG132, indicating that PHB2 similarly stabilizes AURKA.

In summary, AURKA and PHB2 physically interact and positively regulate each other at both the transcriptional and post-translational levels. This positive feedback loop may play a key role in sustaining Sotorasib resistance in NSCLC cells.

### Omics data analysis reveals AURKA knockdown inhibits PI3K-Akt pathways in Calu-1-SR cells

Based on transcriptomic profiling of Calu-1-SR cells and their isogenic AURKA-knockdown counterparts, GSEA revealed that the PI3K-Akt signaling pathway was significantly suppressed in AURKA-knockdown Calu-1-SR compared to Calu-1-SR samples (NES = −1.58, *p* < 0.001, adjusted *p* < 0.05; Fig. 5D). This suppression was consistently observed across both analytical approaches, with ORA identifying the PI3K-Akt pathway as one of the top downregulated pathways (Fig. 5E). The enrichment plot demonstrated a distinct depletion of PI3K-Akt-related genes across the ranked DEG list (Fig. 5F), with the majority of pathway genes located at the lower end of the ranked list. Additionally, GSEA identified significant suppression of related oncogenic pathways, including focal adhesion, chemokine signaling pathway, and Ras signaling pathway (Fig. 5E, F).

These results demonstrate that AURKA knockdown robustly inhibits the PI3K-AKT signaling pathway, suggesting a critical role for AURKA in regulating this oncogenic pathway.

The Western blot (WB) results showed that the activity of the AKT signaling pathway was downregulated in AURKA-SH cells treated with Sotorasib. Re - upregulating the expression of PHB2 in AURKA - SH cells could reverse the down - regulated expression of the AKT signaling pathway(Fig. 5G).

These results indicate that PHB2 interacts with AURKA to modulate Sotorasib sensitivity via the AKT pathway

### PHB2 upregulation is critical for AURKA-mediated Sotorasib resistance

**Fig. 6:**
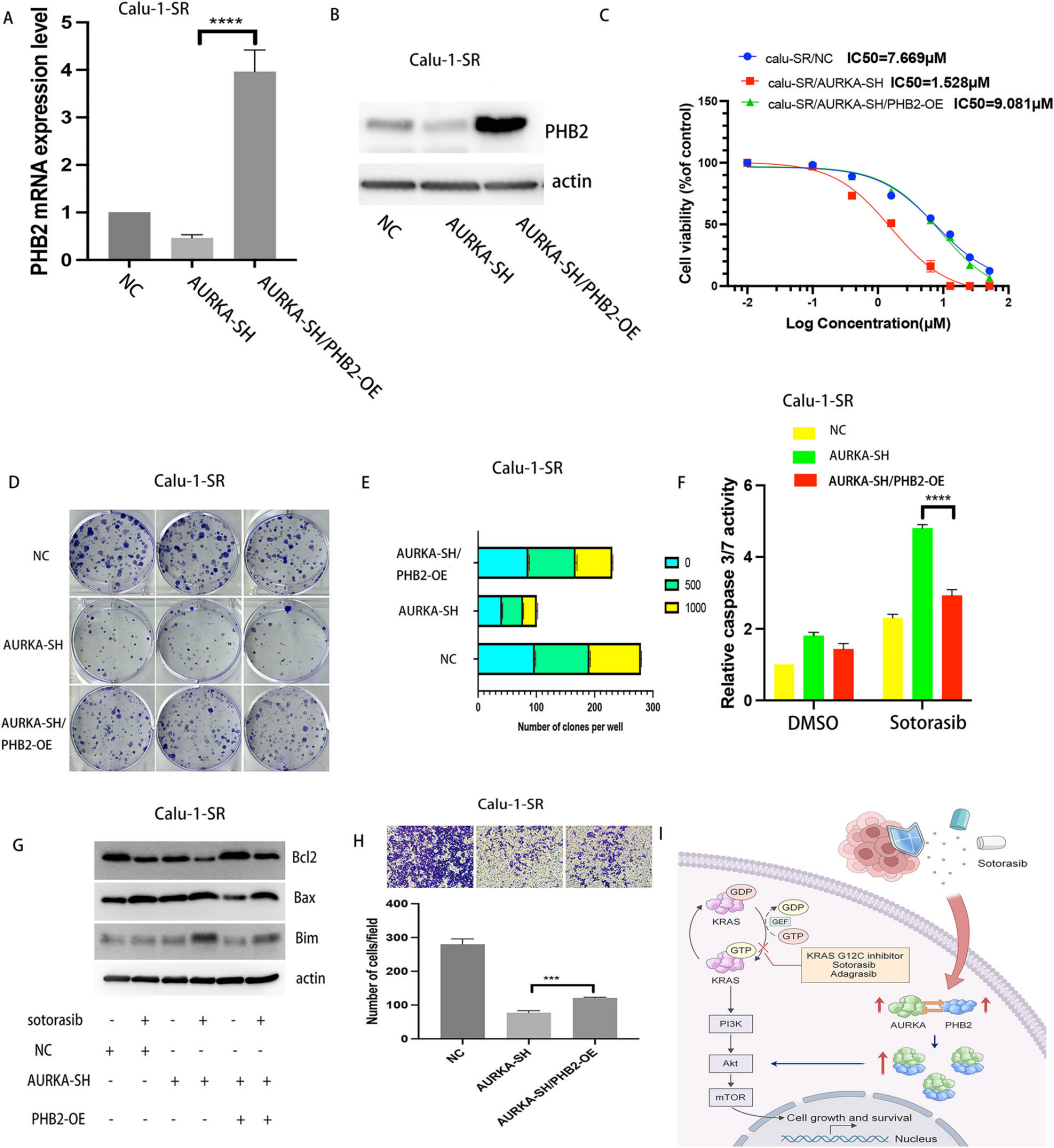
PHB2 upregulation is critical for AURKA-mediated Sotorasib resistance. **A**, **B** qPCR and WB were employed to measure the expression level of PHB2 in Calu-1-SR/NC, Calu-1-SR/AURKA-SH and Calu-1-SR/AURKA-SH/PHB2-OE cells. **C** MTS was used to detect the cell viability of Calu-1-SR/NC, Calu-1-SR/AURKA-SH and Calu-1-SR/AURKA-SH/PHB2-OE cells after treatment with Sotorasib, and IC50 was calculated by SPSS27. **D**, **E** Colony formation assay was used to detect the cell proliferation of Calu-1-SR/NC, Calu-1-SR/AURKA-SH and Calu-1-SR/AURKA-SH/PHB2-OE cells after treatment with Sotorasib. **F** Assay for Caspase 3/7 activity was employed to measure the apoptosis of Calu-1-SR/NC, Calu-1-SR/AURKA-SH and Calu-1-SR/AURKA-SH/PHB2-OE cells treated with Sotorasib. **G** Western blot was used to detect the expression of apoptosis-related proteins Bcl-2, Bax and Bim in Calu-1-SR/NC, Calu-1-SR/AURKA-SH and Calu-1-SR/AURKA-SH/PHB2-OE cells treated with Sotorasib. **H** Transwell assay was used to determine the migration ability of Calu-1-SR/NC, Calu-1-SR/AURKA-SH and Calu-1-SR/AURKA-SH/PHB2-OE cells. **I** Schematic diagram illustrating AURKA/PHB2-mediated regulation of KRAS G12C resistance to Sotorasib.

To further investigate the role of PHB2 in AURKA-associated Sotorasib resistance, a rescue experiment was conducted by overexpressing PHB2 in Calu-1-SR cells with AURKA knockdown. qPCR and Western blot analyses confirmed that PHB2 expression was significantly elevated in Calu-1-SR/AURKA-SH/PHB2-OE cells compared to Calu-1-SR/AURKA-SH controls (Fig. 6A, B).

AURKA knockdown enhanced Sotorasib sensitivity, and this enhancement was partially reversed by PHB2 overexpression via lentiviral transduction. Specifically, the IC_50_ values of Sotorasib were 7.669 uM for Calu-1-SR/NC, 1.528 uM for Calu-1-SR/AURKA-SH, and 9.081 uM for Calu-1-SR/AURKA-SH/PHB2-OE(Fig. 6C). Demonstrating that PHB2 plays a key role in restoring drug resistance.

In the clonogenic assay, PHB2 overexpression significantly enhanced the colony formation ability of AURKA-deficient cells under Sotorasib treatment (Fig. 6D, E). Furthermore, PHB2 overexpression reduced caspase-3/7 activity, indicating decreased apoptosis (Fig. 6F). Western blot analysis revealed increased expression of the anti-apoptotic protein Bcl-2, alongside decreased levels of the pro-apoptotic proteins Bax and Bim, in PHB2-overexpressing, AURKA-knockdown cells (Fig. 6G). Transwell migration assays revealed that PHB2 overexpression also enhanced the migratory capacity of Calu-1-SR/AURKA-SH cells (Fig. 6H). Collectively, these findings highlight the pivotal role of PHB2 in AURKA-driven resistance to Sotorasib by promoting cell survival and migration, and suppressing apoptosis in NSCLC cells.

### Pharmacological inhibition of AURKA overcomes Sotorasib resistance in vitro and in vivo

Given the elevated expression of AURKA and PHB2 in Sotorasib-resistant cells, we next investigated whether pharmacological inhibition of AURKA could restore sensitivity to Sotorasib. We employed Alisertib (MLN8237), a selective oral Aurora A kinase inhibitor (IC_50_ = 132 nM), known to disrupt mitotic spindle formation and mitotic arrest. Additionally, Alisertib has been reported to promote apoptosis and autophagy via modulation of the AKT/mTOR/AMPK/p38 signaling pathways [22, 23].

**Fig. 7:**
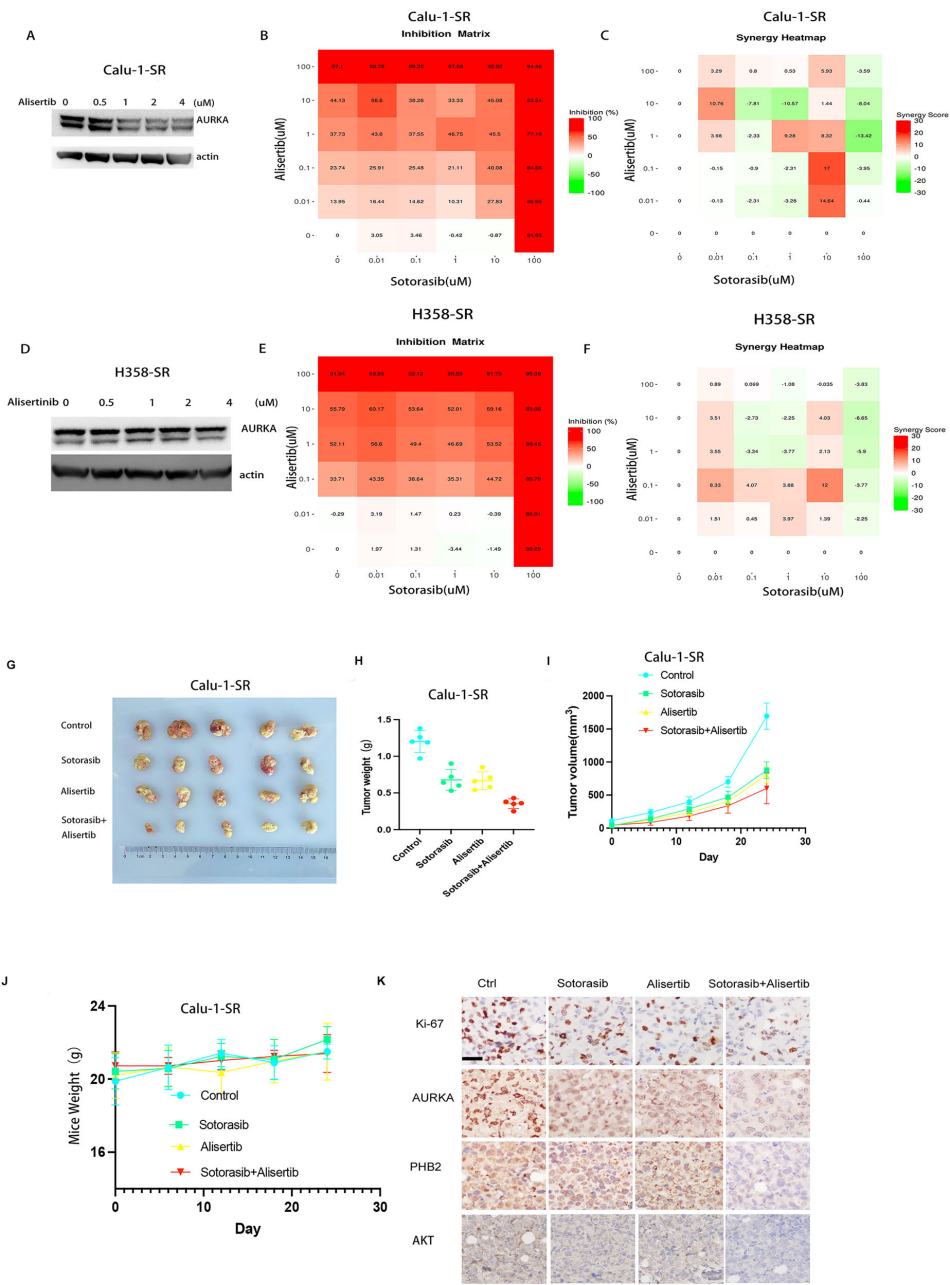
Pharmacological inhibition of AURKA overcomes Sotorasib resistance in vitro and in vivo. **A**, **D** H358-SR or Calu-1-SR cells underwent treatment with the specified concentrations of the AURKA inhibitor Alisertib for 48 h, and the expression level of AURKA measured via Western Blot analysis. **B**, **E** Calu-1-SR cells were treated with Sotorasib and Alisertib for 48 h; cell viability and inhibition rate were assessed using MTS. **C**, **F** The score of the drug synergy experiment was determined by the Bliss model and displayed as a heatmap. **G**, **I** 5 × 10^6^ Calu-1-SR cells were injected subcutaneously into nude mice. After allowing palpable tumors to develop for 7 days, the mice were randomly assigned to four groups. These groups were treated with vehicle control (0.01% DMSO in PBS), Sotorasib (30 mg/kg/d), Alisertib (30 mg/kg/d), or a combination of Sotorasib and Alisertib administered every other day (*n* = 6 per group). **G** The tumor size was measured at specified time intervals and calculated. The tumor volume was computed using the formula: *V* = 1/2 × larger diameter × (smaller diameter)^2^. Growth curves were then plotted using the average tumor volume within each experimental group at the set time points. **H** At the conclusion of the treatment, the tumors were excised. **I** The weights of the tumors were measured. **J** Sotorasib and/or Alisertib administration does not influence the weight of the mice. **K** Calu-1-SR xenograft tissues obtained from mice were fixed, sliced into sections, and then mounted on glass slides. Tumor samples were processed for IHC staining using Abs against Ki-67, AURKA, and PHB2.

Western blot analysis showed that treatment of Calu-1-SR and H358-SR cells with increasing concentrations of Alisertib resulted in a dose-dependent reduction of AURKA protein expression, confirming the drug’s inhibitory effect (Fig. 7A, D). Both Sotorasib and Alisertib alone exhibited concentration-dependent cytotoxicity. At 100 μM, Sotorasib achieved maximum inhibition rates of 87.1% in Calu-1-SR and 91.94% in H358-SR, while Alisertib reached 84.93% and 90.29%, respectively. Notably, co-treatment with Alisertib and Sotorasib further enhanced the antiproliferative effect.

To evaluate synergy, we applied the Bliss independence model. The highest Bliss synergy scores were 12 in Calu-1-SR and 17 in H358-SR, indicating a synergistic interaction. The strongest synergy occurred with 0.1 μM Alisertib combined with 10 μM Sotorasib in both cell lines (Fig. 7B, C, E, F).

These results indicate that targeting AURKA pharmacologically with Alisertib can significantly enhance the efficacy of Sotorasib and overcome resistance in NSCLC cells.

To validate the in vitro findings in a physiological context, we assessed the therapeutic efficacy of Sotorasib and Alisertib combination treatment using a xenograft model in nude mice. H358-SR cells were subcutaneously implanted into five-week-old nude mice. Once measurable tumors had developed, the animals were randomly assigned to four groups and administered Sotorasib (30 mg/kg/day), Alisertib (30 mg/kg/day), or a combination of both drugs [24, 25].

In line with our in vivo observations, monotherapy with either Sotorasib or alisertib led to modest tumor growth inhibition. In contrast, combined treatment significantly suppressed tumor progression (Fig. 7G–I). Importantly, there were no significant differences in body weight among the groups, suggesting that the treatments were well-tolerated and did not cause overt toxicity (Fig. 7J).

Immunohistochemical analysis further revealed that combined treatment markedly reduced the expression of Ki-67, AURKA, and PHB2 in tumor tissues (Fig. 7K), supporting the proposed mechanism of action.

These results provide in vivo evidence that pharmacological inhibition of AURKA can effectively overcome Sotorasib resistance in NSCLC, highlighting the potential of this combinatorial strategy for future therapeutic development. Schematic diagram of AURKA/PHB2-mediated regulation of KRAS G12C resistance to Sotorasib: In Sotorasib-resistant NSCLC cells, AURKA expression is upregulated. AURKA binds to PHB2 and establishes a positive feedback loop through both transcriptional and translational regulation. This interaction sustains Sotorasib resistance in KRAS ^G12C^-mutant NSCLC cells by modulating the PI3K/AKT signaling pathway.

## Discussion

This study aimed to investigate the underlying mechanisms of Sotorasib resistance in NSCLC. Our findings revealed that in Sotorasib-resistant cells, AURKA expression was upregulated, along with its binding partner PHB2, resulting in a positive feedback loop at both the transcriptional and post-translational levels. Through modulation of these genes, either by overexpression or knockdown, we observed that overexpressing AURKA or PHB2 specifically enhanced Sotorasib resistance in H358 and Calu-1 cells. Conversely, knocking down AURKA or PHB2 increased the sensitivity of cancer cells to Sotorasib. Mechanistically, AURKA promoted drug resistance in lung cancer cells through the PHB2/PI3K/AKT signaling cascade, underscoring AURKA’s critical role in the observed Sotorasib insensitivity in NSCLC cells.

Resistance to KRAS ^G12C^ inhibitors, such as Sotorasib, has been attributed to several mechanisms, including changes in the KRAS protein, activation of bypass signaling pathways, and factors within the tumor microenvironment [4]. However, the precise mechanisms remain incompletely understood. Our data suggest that AURKA upregulation induced by Sotorasib is independent of the previously known resistance mechanisms, positioning AURKA as a key determinant in the emergence of resistance to Sotorasib. Activation of the KRAS/Raf/MEK/ERK pathway and PI3K–AKT–mTOR pathway is a primary oncogenic driver of cancer cell proliferation and migration [26]. When Sotorasib inhibits this pathway, cancer cells adapt by activating alternative signaling routes to maintain survival. Our findings demonstrate that AURKA upregulation activates the PHB2/PI3K/AKT pathway, enabling cancer cells to bypass the KRAS blockade and restore their malignant behavior.

AURKA is a serine/threonine kinase composed of 403 amino acids [27]. Its dysregulated expression is implicated in various tumor types. AURKA inhibition enhances the effectiveness of treatments in cancers such as diffuse large B-cell lymphoma by attenuating β-Catenin and the RAS/ERK1/2 pathway [15]. In alignment with these findings, we observed that blocking the KRAS pathway with Sotorasib led to AURKA upregulation, which in turn activated PHB2/PI3K/AKT signaling, compensating for the inhibited KRAS oncogenic pathway. Additionally, overexpression of AURKA in sensitive cell lines like H358 and Calu-1 selectively conferred Sotorasib resistance, while silencing AURKA enhanced sensitivity to Sotorasib. These results suggest that AURKA may serve as a “switch”, modulating the PHB2/PI3K/AKT pathway to sustain cancer cell proliferation and migration under KRAS pathway inhibition.

PHB2 is located at the 12p13.31 region on human chromosome 12, encoding a multifunctional protein that is highly conserved. In some cancers, PHB2 influences growth factor receptor signaling. For example, in gastric cancer, it promotes SHIP2 ubiquitination via NEDD4, affecting AKT signaling [28]. Alterations in these pathways contribute to tumor cell proliferation, survival, and migration. PHB2 has also been implicated in drug resistance. In certain breast cancer subtypes, overexpression of PHB2 is associated with resistance to endocrine therapy. Furthermore, AURKA interacts with MAP1LC3 and PHB2 in mitochondria to initiate mitophagy [29]. Our study supports a direct interaction between PHB2 and AURKA, with positive feedback regulation at both the transcriptional and post-translational levels. Regulation of PHB2 expression in resistant cells reversed the Sotorasib resistance observed with AURKA knockout, indicating that the AURKA/PHB2/PI3K/AKT signaling pathway plays a critical role in the development of Sotorasib resistance.

The combination therapy approach is critical in overcoming drug resistance. We observed elevated levels of AURKA and PHB2 in Sotorasib-resistant cells and investigated the potential for AURKA inhibition to sensitize these cells to Sotorasib. Alisertib (MLN 8237), a selective Aurora A kinase inhibitor, binds to Aurora A kinase and induces the formation of abnormal mitotic spindles, thereby accumulating cells in mitosis [30]. In our experiments, combined treatment with Sotorasib and alisertib significantly enhanced cell proliferation inhibition in Calu-1-SR and H358-SR cells. The highest synergy was observed with alisertib at 0.1 μM and Sotorasib at 10 μM. Furthermore, when tested in a xenograft nude mouse model, the combination therapy further strengthened the hypothesis that pharmacological inhibition of AURKA can overcome Sotorasib resistance in NSCLC.

## Materials and methods

### Data collection and processing

For prognostic analysis, six lung cancer datasets with survival information were collected: GSE157009 [31], GSE30219 [32], GSE41271 [33], GSE42127 [34], and TCGA-LUAD. To identify tumor-adjacent differentially expressed genes (DEGs), eleven datasets containing paired tumor and adjacent normal tissue samples were obtained: GSE10072 [35], GSE140797 [36], GSE18842 [37], GSE19804 [38], GSE21933 [39], GSE30219 [32], GSE31210 [40], GSE33479 [41], GSE33532 [42], GSE43458 [43], and TCGA-LUAD. Single-cell RNA sequencing (scRNA-seq, GSE137912 [18]) data from three LUAD cell lines (H2122, H358, SW1573) and bulk RNA-seq data from two GEO datasets (GSE204752 [44], GSE278656 [45]) were analyzed to characterize Sotorasib resistance-associated gene signatures. these data are available and free from the public databases

### Identification of prognostic risk genes

Cox proportional hazards regression analysis was performed on five independent datasets to identify genes significantly associated with overall survival (OS). The survival package in R (v4.3.1) was employed, with hazard ratios (HR) and 95% confidence intervals calculated for each gene. Genes with *p* < 0.05 and HR > 1 were defined as prognostic risk genes. Kaplan-Meier (KM) survival curves were generated for key gene expression levels (high vs. low) across all datasets using the survival and survminer packages. Patients were stratified by median gene expression and Log-rank tests were applied to assess survival differences.

### Differential gene expression analysis

The limma package in R was employed to compare tumor versus adjacent normal tissue transcriptomes across 11 datasets. Raw microarray data were normalized using quantile normalization, while RNA-seq data were log2-transformed. Differentially expressed genes (DEGs) were identified with thresholds of |log2-fold change (FC)| ≥ 0.5 and adjusted *p* < 0.01.

### Sotorasib resistance gene profiling

For scRNA-seq data, the Seurat package (v4.3.0) was used for quality control, dimensionality reduction, and clustering. Resistance-associated genes were identified via differential expression analysis (FindAllMarkers function) comparing resistant clusters to controls. For bulk RNA-seq, DEGs between resistant and sensitive samples were determined using limma, with thresholds of |log2FC | ≥ 0.5 and adjusted *p* < 0.05.

### Establishment of Sotorasib-resistant cell lines

The KRAS G12C-mutant NSCLC cell lines Calu-1 and H358 were purchased from the American Type Culture Collection (ATCC). Cells were cultured in RPMI-1640 medium supplemented with 10% fetal bovine serum (FBS; GIBCO, Cat. No. 10099233), 100 U/mL penicillin, and 100 µg/mL streptomycin. All cell cultures were maintained at 37 °C in a humidified incubator with 5% CO₂.

To generate Sotorasib-resistant (SR) cell lines, H358 and Calu-1 cells were continuously exposed to increasing concentrations of Sotorasib (MCE, Shanghai, China; Cat. No. HY-114277), ranging from 0 to 51,200 nM in stepwise increments: 0, 100, 200, 400, 800, 1600, 3200, 6400, 12,800, 25,600, and 51,200 nM. After 25 cell passages over a period of 4 months, Sotorasib was withdrawn from the culture medium. Surviving cells capable of proliferating at 50,000 nM Sotorasib were designated as Sotorasib-resistant cell lines, H358-SR and Calu-1-SR.

### Immunohistochemistry (IHC)

A total of 25 paired KRAS G12C-positive and 50 wild-type non-small cell lung cancer (NSCLC) human tissue samples were collected from Fujian Cancer Hospital (Fuzhou, China) between September 2020 and April 2024 for immunohistochemistry (IHC) analysis. All samples were obtained from surgical resections of NSCLC patients and reviewed independently by two pathologists at Fujian Cancer Hospital. The study was approved by the Research Ethics Committee of the Fujian Provincial Cancer Hospital, China. Informed consent from all patients was obtained prior to the study.

For IHC analysis, tissues were fixed in 4% paraformaldehyde (PFA), embedded in paraffin, and sectioned at a thickness of 4 μm. The sections were baked at 58 °C for 24 h, deparaffinized in xylene, rehydrated through a graded ethanol series, treated with 0.3% hydrogen peroxide at 37 °C for 15 min to quench endogenous peroxidase activity, and subjected to antigen retrieval. Sections were then blocked with 1% BSA in PBS (Sangon, Shanghai, China). Sections were incubated overnight at 4 °C with primary antibodies against AURKA (1:100; MCE, Cat. No. HY-P80971), PHB2 (1:100; MCE, Cat. No. HY-P82103), and Ki-67 (1:500; CST, Cat. No. #9449), followed by incubation with a secondary antibody and horseradish peroxidase (HRP)-conjugated streptavidin for 15 min. Immunoreactivity was visualized using DAB, and nuclei were counterstained with hematoxylin. Protein staining intensity was graded on a semiquantitative scale: 0 (no staining relative to background), 1 (weak), 2 (moderate), and 3 (intense). The H-score is used to generate a comprehensive score by combining the percentage of positive cells and the staining intensity.

The formula is:

$${{\rm{H}}}_{-{\rm{score}}}=\Sigma ({{\rm{P}}}_{{\rm{i}}\times {\rm{i}}})\times 100$$

where Pi represents the proportion of positive cells with a certain staining intensity, and i represents the grading of staining intensity.

### Generation of AURKA-overexpressing and AURKA-knockdown cell lines

Lentiviral vectors were obtained from Hanheng Biotechnology Company. Ltd. (Beijing, China). To establish AURKA-overexpressing cell lines, cells were transduced with the HBLV-AURKA expression construct (pHBLV-CMV-mcs-3flag-EF1-ZsGreen-T2A-PURO), which carries the human AURKA gene. The empty vector served as the negative control.

For AURKA knockdown, cells were transduced with one of three shRNA constructs (HBLV-AURKA-shRNA1/2/3). The pHBLV-U6-MCS-CMV-ZsGreen was used as the negative control. H358, H358-SR, Calu-1, and Calu-1-SR cells were plated at a density of 5 × 10⁵ cells per well and infected at a multiplicity of infection (MOI) of 10 in the presence of 6 µg/mL polybrene. Stable transductants were selected using 2 µg/mL puromycin over a 2-week period.

The resulting experimental groups were as follows:

- H358-SR/Vector
- H358-SR/AURKA-SH
- H358/NC
- H358/AURKA-SH
- Calu-1-SR/NC
- Calu-1-SR/AURKA-SH
- Calu-1/NC
- Calu-1/AURKA-SH

(*Note: “-OE” and “-SH” indicate AURKA overexpression and shRNA-mediated knockdown, respectively*.)

### Quantification of AURKA and PHB2 mRNA expression

Quantitative real-time PCR (qRT-PCR) was utilized to measure the mRNA expression levels of AURKA and PHB2 in NSCLC cell lines (H358, Calu-1, H358-SR, Calu-1-SR). Total RNA was isolated from cell lines using TRIzol Reagent (Invitrogen, Grand Island, NY, USA) according to the manufacturer’s instructions. One microgram of total RNA was reverse-transcribed into cDNA using M-MuLV reverse transcriptase (Promega, Madison, WI, USA). PCR amplification was conducted with the SYBR Green detection system (Roche, Switzerland). Primer sequences for AURKA and PHB2 are listed in Supplementary Table 1. The PCR cycling conditions were conducted as previously described [11, 12].

Each qPCR reaction was performed in triplicate. Relative gene expression was calculated using the 2^−ΔΔCt^ method, with GAPDH as the internal control.

### Protein levels of AURKA, PHB2, and AKT signaling pathway components

The protein expression levels of AURKA, BAX, Bcl-2, PHB2, AKT, and Bim were evaluated by western blot. The following antibodies were used:

- AURKA (1:1000; CST, Danvers, MA, USA; Cat. No. 91590)
- BAX (1:1000; CST; Cat. No. 2772)
- Bcl-2 (1:1000; CST; Cat. No. 15071)
- PHB2 (1:1000; CST, Danvers, MA, USA; Cat. No. 14085)
- p-AKT (CST; Cat. No. 4046)
- AKT (CST; Cat. No. 9272)
- Bim (1:1000; CST; Cat. No. 2819)

### Effects of Sotorasib on cell proliferation and IC50 determination

Cells were plated at a density of 5000 cells per well in a 96-well microplate and incubated overnight. The following day, cells were treated in triplicate with increasing concentrations of Sotorasib (0, 200, 400, 800, 1,600, 3,200, 6,400, 12,800, 25,600, and 51,200 nM).

After 72 h of incubation, 20 µL of MTS reagent (Promega, Madison, WI, USA; Cat. No. G3582) and 100 µL of complete medium were added to each well. Plates were incubated at 37 °C for an additional 2 h. Wells containing only medium with serum served as background controls.

Absorbance was measured at 490 nm using a Bio-Rad microplate reader (Model 680, Hercules, CA, USA). The proliferation rates were normalized to untreated controls, and IC₅₀ values were calculated using SPSS version 27.0. All experiments were conducted in triplicate.

### Colony formation assay

Digest Calu-1, Calu-1-SR, H358, and H358-SR cells in the logarithmic growth phase with trypsin and resuspend them into single - cell suspensions. Then, inoculate the cells at a density of 500 cells per well in a 6-cm culture dish. After the cells are seeded and adhere to the dish, treat the cells with different concentrations of Sotorasib (0, 500, 1000 nM). Place the culture dishes in an incubator at 37 °C with 5% CO₂ for static culture. Replace the fresh culture medium containing Sotorasib every 3–4 days during the culture period. After 14 days of culture, discard the culture medium and wash the cells carefully twice with PBS. Fix the cells with methanol for 15 min, and then stain them with 0.1% crystal violet solution for 30 min. Finally, slowly wash away excess staining solution with running water, then air-dry the culture dishes at room temperature. Use ImageJ software to manually count the colonies with more than 50 cells. Set at least three replicates for each group of experiments, and repeat all experiments independently three times.

### Caspase 3/7 activity assay

Caspase-3/7 activity was evaluated using the Caspase-Glo^®^ 3/7 Assay Kit (Promega, Cat. No. G8090) in accordance with the manufacturer’s protocol. The reagent was equilibrated to room temperature and thoroughly mixed before use.

Calu-1, H358, Calu-1-SR, and H358-SR cells were treated with 0 or 1 µM Sotorasib for 24 h in white-walled 96-well plates. After incubation, 100 µL of Caspase-Glo^®^ 3/7 reagent was added per well. Plates were gently shaken (300–500 rpm) for 30 s and incubated for 1 h at room temperature. Luminescence intensity was measured using a luminometer.

### Immunofluorescence analysis

Cells were fixed in 4% paraformaldehyde at 37 °C for 15 min and permeabilized with 0.2% Triton X-100 (Sigma-Aldrich, T8787) for 15 min at room temperature and then blocked to reduce non-specific binding.

Cells were incubated with primary antibodies overnight at 4 °C, followed by incubation with appropriate secondary antibodies at room temperature for 1 h. After washing, nuclei were stained with DAPI. The colocalization of AURKA and PHB2 was visualized using an Olympus FV1000 laser scanning confocal microscope.

### Co-immunoprecipitation (Co-IP) analysis

Co-IP was carried out using the Protein A Magnetic Beads Immunoprecipitation Kit (Beyotime, Shanghai, China), according to the manufacturer’s protocol. Cells were rinsed with ice-cold PBS and lysed in buffer containing protease inhibitors on ice. Lysates were centrifuged at 12,000 × *g* for 10 min at 4 °C, and the resulting supernatants were precleared with Protein A Magnetic Beads for 30 min at 4 °C.

Following preclearing, the supernatants were incubated overnight at 4 °C with either specific primary antibodies or a control IgG antibody to facilitate immune complex formation. Afterward, the samples were incubated with Protein A Magnetic Beads for an additional 2 h at 4 °C. Immunocomplexes were washed, resuspended, and boiled in 1 × SDS loading buffer at 95 °C for 10 min. The samples were subsequently analyzed by Western blot. IgG was used as a negative control.

### Transwell cell invasion assay

Transwell invasion assays were conducted as previously described [46]. Migrated cells on the Transwell membranes were visualized using a light microscope (Olympus, Japan) and quantified using ImageJ software. Five random fields per sample were analyzed, and results were expressed as mean ± SD. Each experiment was performed in triplicate to ensure reproducibility.

### Promoter luciferase assays

Promoter regions of AURKA and PHB2, covering 200 bp, 500 bp, and 1000 bp upstream sequences, were cloned into the pGL3-basic luciferase reporter vector. All constructs were validated by sequencing.Calu-1/NC, Calu-1/AURKA-SH, and Calu-1/PHB2-OE cells were transfected with pGL3-AURKA-p500, pGL3-AURKA-p1000, pGL3-PHB2-p500, or pGL3-PHB2-p1000 using X-tremeGENE HP DNA Transfection Reagent (Roche, Basel, Switzerland; Cat. No. 6366236001). Firefly luciferase activity was measured using the Dual-Luciferase Reporter Assay Kit (Promega, Madison, WI, USA). Firefly luciferase signals were normalized to Renilla luciferase activity to correct for transfection efficiency.

### Transcriptomic data analysis of AURKA gene knockdown in Calu-1-SR

To investigate the functional enrichment of DEGs between control (Calu-1-SR) and AURKA-knockdown Calu-1-SR cells, we performed pathway analysis using Over-Representation Analysis (ORA) and Gene Set Enrichment Analysis (GSEA). RNA-seq data from six tumor samples (three Calu-1-SR samples versus three AURKA-knockdown Calu-1-SR samples) were analyzed using DESeq2 in R to identify DEGs with thresholds of |fold change| ≥ 1.5 and *p* < 0.05. Subsequently, ORA was conducted using the clusterProfiler package (v4.3.1) in R to identify significantly enriched pathways from the KEGG database, with multiple testing correction applied (adjusted *p* < 0.05). GSEA was performed using the fgsea package to assess directional overrepresentation of predefined gene sets, with normalized enrichment scores (NES) and false discovery rate (FDR)-adjusted *p* values calculated. Pathways with NES < 0 and FDR-adjusted *p* < 0.05 were defined as significantly suppressed.

### Animal experiments

Five-week-old female BALB/c nude mice were purchased from Shanghai Jihui Laboratory Animal Breeding Co., Ltd. A total of 5 × 10⁵ Sotorasib-resistant Calu-1-SR cells were injected subcutaneously into the mice. Once tumors reached an average volume of 50 mm³, the mice were randomized into four treatment groups (*n* = 6 per group):

- PBS (vehicle control)
- Sotorasib (30 mg/kg, i.g.)
- Alisertib (30 mg/kg, i.g.)
- Sotorasib + Alisertib (both at 30 mg/kg, i.g.)

Treatments were administered every other day. Tumor volumes were calculated using the formula: Volume = (Length × Width²) × 0.5.

At the end of the treatment period, tumors were excised, weighed, and processed for fixation and paraffin embedding. All animal procedures complied with the Institutional Animal Care and Use Committee (IACUC) of Fujian Provincial Hospital and conducted in accordance with ethical guidelines and approved protocols.

### Molecular docking analysis

To assess the potential direct interaction between AURKA and PHB2, molecular docking analysis was performed. The full-length three-dimensional structures of human AURKA and PHB2 were obtained from the AlphaFold Protein Structure Database and submitted to the ClusPro protein–protein docking server for rigid-body docking using default parameters.

ClusPro applies an FFT-based algorithm to sample and cluster docking conformations, which are ranked according to binding energy and cluster size. The top-ranked model with the lowest binding energy and the largest cluster size was selected for further analysis. Structural visualization and interaction analysis were conducted using PyMOL. Hydrogen bonds were defined using standard geometric criteria (donor–acceptor distance ≤ 3.0 Aand donor–hydrogen–acceptor angle > 120°).

### Statistical analysis

Data are presented as mean ± standard deviation (SD). Student’s *t* test was applied for comparisons between two groups, while one-way ANOVA was used for comparisons involving multiple groups. *P* *value* *<* 0.05 was considered statistically significant.

## Supplementary information

Supplementary Information

Full and uncropped western blots

## Data Availability

The data supporting the findings of this study are available from the corresponding author upon reasonable request. All raw sequencing data have been deposited in the Gene Expression Omnibus (GEO) repository and are publicly accessible via the accession code GSE316749.
