# Supplementary material for: AURKA/PHB2 signaling drives acquired resistance to KRAS G12C inhibitors in KRAS G12C-mutant NSCLC
Source: Cell Death Discov. 2026 Apr 25;12:273. doi: 10.1038/s41420-026-03080-4 (PMC13247048; doi:10.1038/s41420-026-03080-4)
Supplement: Supplementary file 1 — Supplementary Information [file 41420_2026_3080_MOESM1_ESM.pdf]

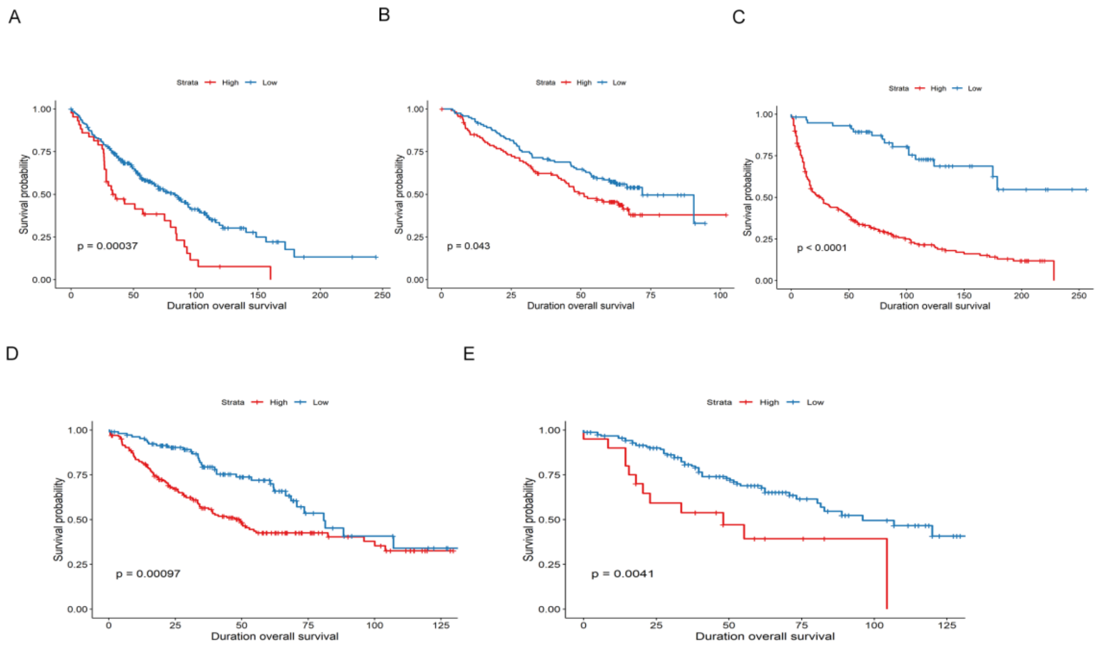

Supplementary Fig. S1 High AURKA expression is associated with poor overall survival in multiple independent lung adenocarcinoma cohorts.

A GSE157009 . **B** GSE157010. **C** GSE30219. **D** GSE41271 **E** GSE42127. In each cohort, patients were stratified into high (red line) and low (blue line) AURKA expression groups based on the optimal cut-off value. The log-rank test was used to calculate p-values. Hazard ratios (HR) with 95% confidence intervals are shown for each dataset.

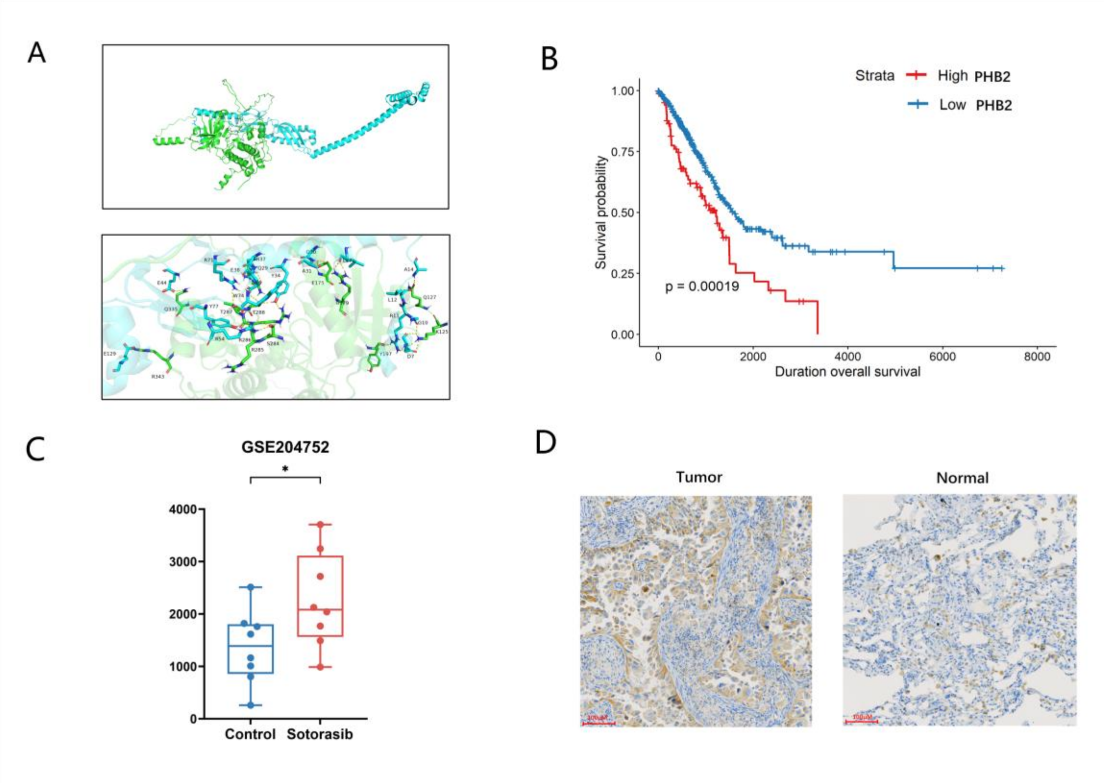

Supplementary Fig.S2 Molecular docking analysis of the AURKA–PHB2 interaction and its association with Sotorasib resistance and survival

A The docked complex of AURKA (green) and PHB2 (blue) is shown. The overall structure and enlarged views of the interaction interface are presented (up). Labeled residues indicate key interacting sites, and yellow dashed lines denote predicted hydrogen bonds stabilizing the AURKA–PHB2 complex (down). Key interacting residues on AURKA included R343, Q335, S284, R285, R286, T287, T288, Q29, E175, R179, K125, Q127, and Y197, while critical residues on PHB2 included E129, E44, Y77, R54, R71, W74, E38, R37, Y34, G30, A31, L27, D7, G10, R11, L12, and A14. These interactions, particularly hydrogen bonds at the docking interface, likely contribute to the structural stability of the AURKA–PHB2 complex.**B** Kaplan-Meier survival curves for TCGA-LUAD patients stratified by PHB2 expression (high vs. low; log-rank p < 0.001). **C** Boxplot showing PHB2 expression levels in Sotorasib-resistant versus sensitive tumors from 16 Kras-G12C/Trp53 genetically engineered mice (**p < 0.01, Wilcoxon test) **D** Immunohistochemical (IHC) expression of PHB2 in tumor tissues versus normal tissues from lung adenocarcinoma patients with KRAS G12C mutation.

Supplementary Table1. The list and sequence of long primers used for RT-qPCR analysis

Gene	Sequence（5’-3’）	Length(bases)
AURKA		
F-primer	AATTCTTCCCAGCGCATTCC	20
R-primer	TGATGCCAGTTCCTCCTCAG	20
PHB2		
F-primer	GCCAAGTTCAATGCCTCACA	20
R-primer	ATTTTCTGCCGCTGTTCCTG	20

Supplementary Table2. Consistently dysregulated DEGs across LUAD datasets

Gene Name
FAM107A
FHL1
SPP1
GPM6A
AOC3
MFAP4
TMEM100
CHRDL1
FMO2
SRPX
CDO1
SULF1
ZBTB16
CYP4B1
SPARCL1
LEPR
WFDC1
GDF10
GREM1
COL10A1
MYH11
LAMP3
TMEM47
MARCO
IL33
CEP55
LMNB1
FAP
HLF
ZWINT
ECT2
C7

Gene Name
DES
IGFBP3
ITGA8
MELK
COL1A1
KNTC1
PRELP
MCM4
TYMS
RFC4
TPX2
COL3A1
CKS1B
CENPF
ASPM
NEK2
CDKN3
GINS1
CNN1
MMP1
KIF14
IL7R
TK1
UBE2C
OGN
KIF2C
BIRC5
SLPI
FANCI
MMP9
SCN7A
CDCA8
SLC7A5

Gene Name
COL5A2
KIF11
TACC3
CENPA
CCNE1
CXCL13
MKI67
SPINK1
EGLN3
NCAPG
TIMP3
EZH2
CHEK1
NDC80
TRIP13
ATAD2
KIF23
CENPE
ARNTL2
PBK
CCNE2
GALNT14
EEF1A2
SLC7A11
AKR1B10
TREM1

Supplementary Table3. Genes associated with sotorasib_resistance

Gene Name
MCM7
RRM2
MCM4
HMGB2
PCNA
TYMS
MCM3
TRIP13
CDCA4
SAPCD2
CDCA5
CDC6
TACC3
RANBP1
DCTPP1
MCM2
CHAF1A
AURKA
ODC1
DDX11
CDC45
RECQL4
MCM10
PGAM5
NCAPH
CDT1
GINS2
FOXM1
RFC4
NEAT1
SHCBP1

Gene Name
TK1
POLE
SKA3
ORC6
STIL
KIF23
SNRPG
RFC3
ASF1B
ALDH3B1
GINS1
EMG1
XRCC3
CDK2
CHAF1B
CENPU
MAGED2
POLR3K
HELLS
TGIF1
E2F1
MAGOHB
NUDT1
RNASEH2A
HAUS8
BRI3BP
TRAIP
BRCA1
FANCG
OIP5
MALAT1
RFC5
DSCC1

**Gene Name**
MCUR1
PSMC3IP
PRIM1
POLE2
CENPH
BORA
BARD1
ING2
GDF15

Supplementary Table4. Cox-identified survival risk genes in LUAD

Gene Name
ACAT2
ADM
AURKA
BUB1
CDCA2
CDCA4
CDCA5
CDCA8
CDKN3
CDT1
CENPA
CENPF
CENPM
DEPDC1
DIAPH3
ECT2
FANCA
GTSE1
HMMR
MCM10
MELK
OIP5
PBK
PLK4
PRC1
RACGAP1
RFC2
RRM2
SNRPA1
TPX2
TTK
ZWILCH
