# Supplementary material for: AURKA/PHB2 signaling drives acquired resistance to KRAS G12C inhibitors in KRAS G12C-mutant NSCLC
Source: Cell Death Discov. 2026 Apr 25;12:273. doi: 10.1038/s41420-026-03080-4 (PMC13247048; doi:10.1038/s41420-026-03080-4)

G

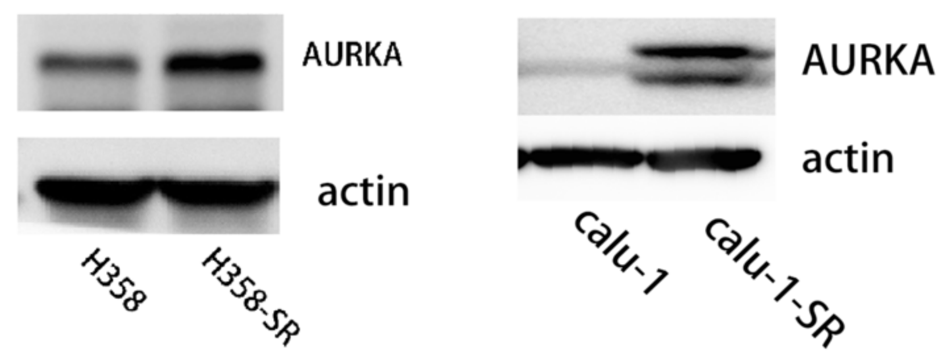

Fig 1 G H358 AURKA

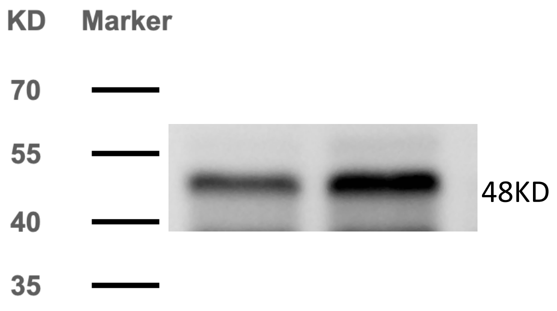

Fig 1 G H358 β-actin

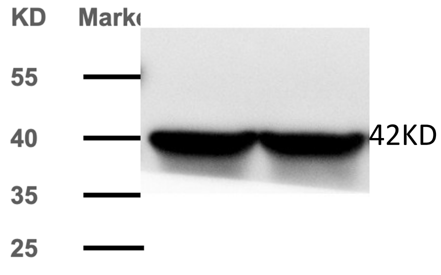

Fig 1 G Calu AURKA

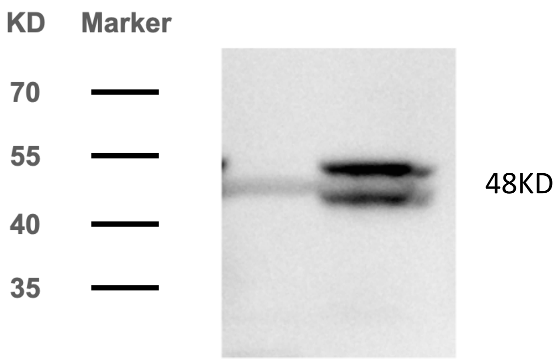

Fig 1 G Calu β-actin

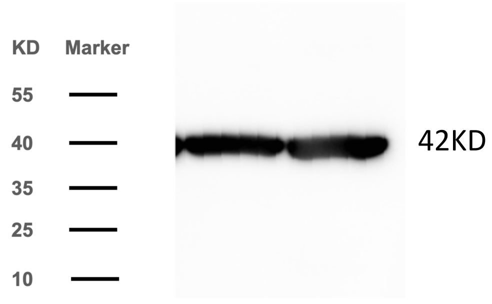

H

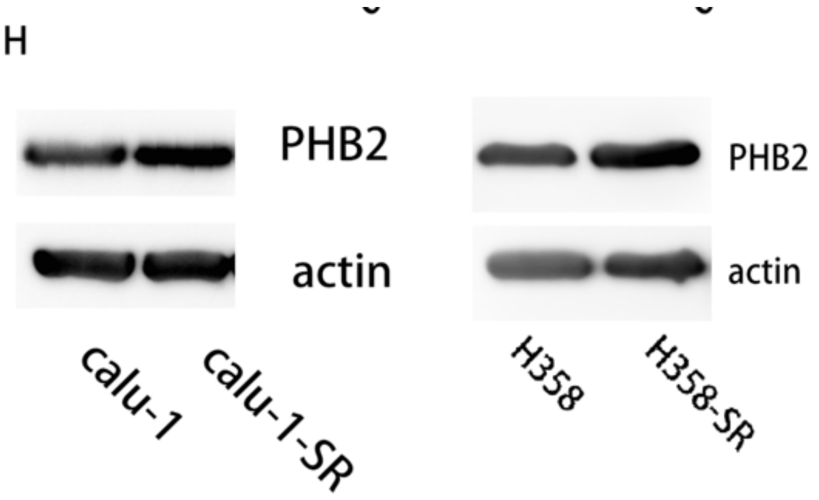

Fig 1 H Calu PHB2

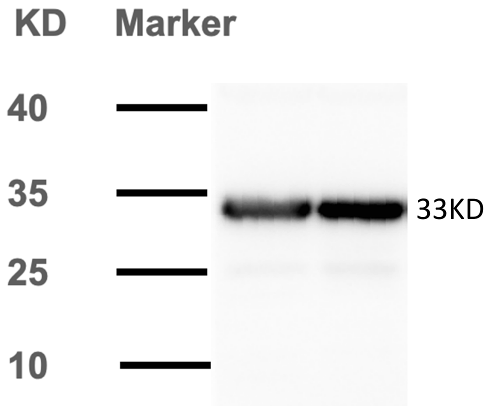

Fig 1 H Calu β-actin

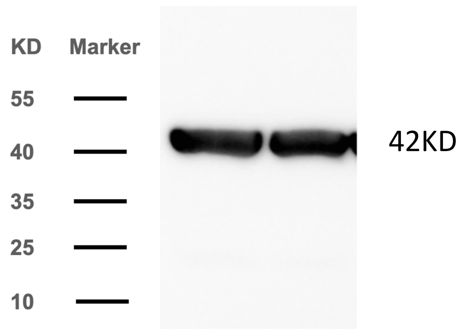

Fig 1 H H358 PHB2

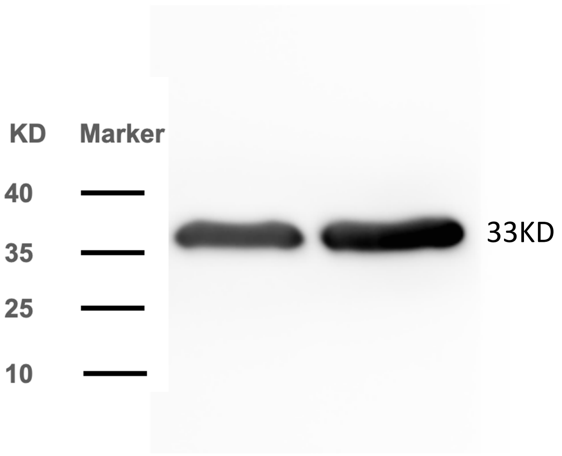

Fig 1H H358 β-actin

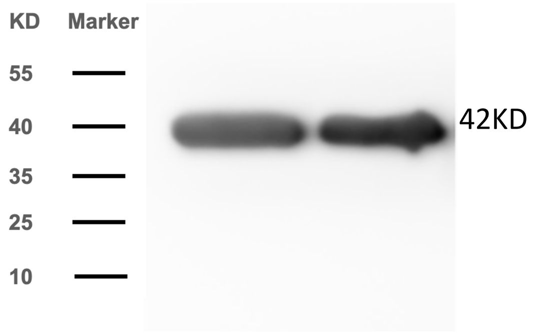

C

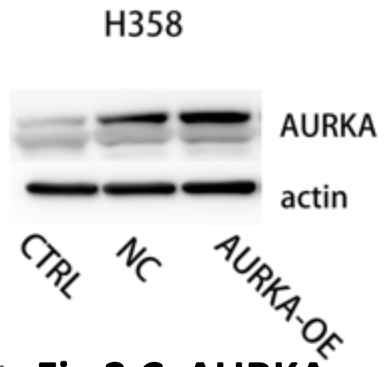

D

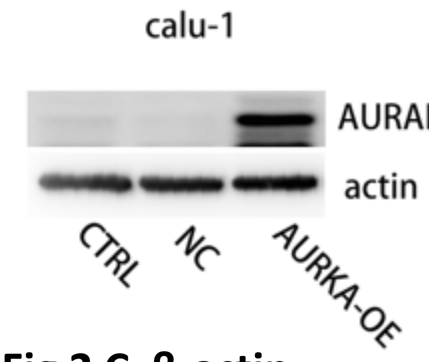

Fig 2 C AURKA

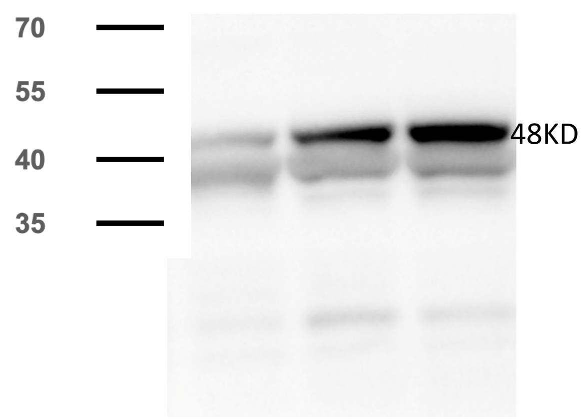

Fig 2 C β-actin

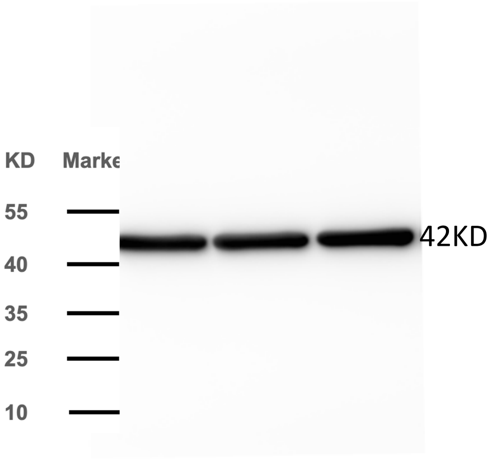

Fig 2 D AURKA

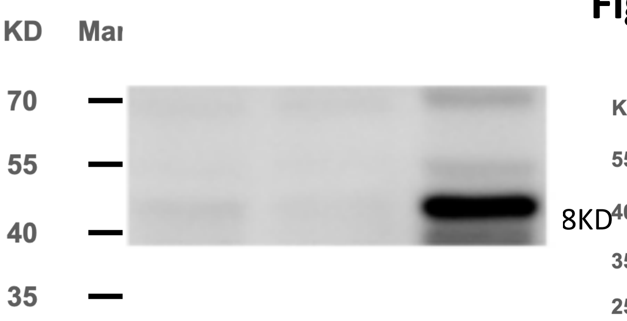

Fig 2 D β-actin

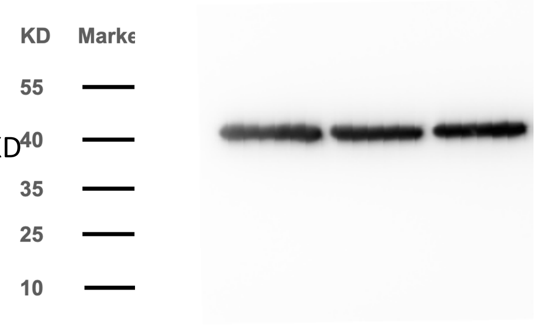

L

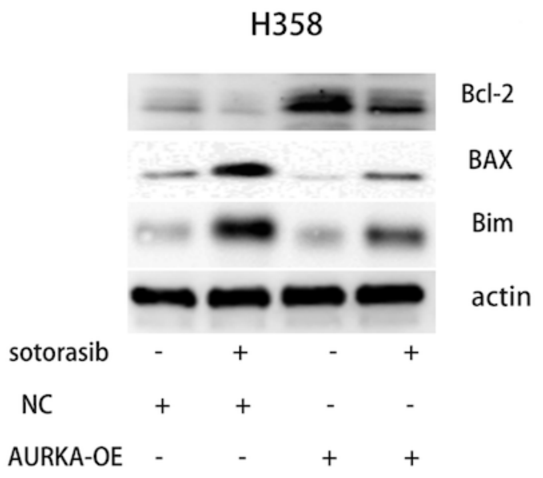

Fig 2 L Bcl-2

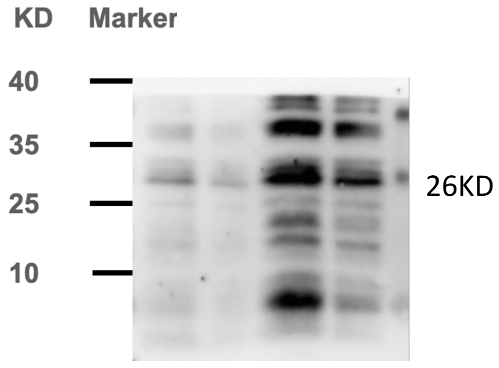

Fig 2 L Bax

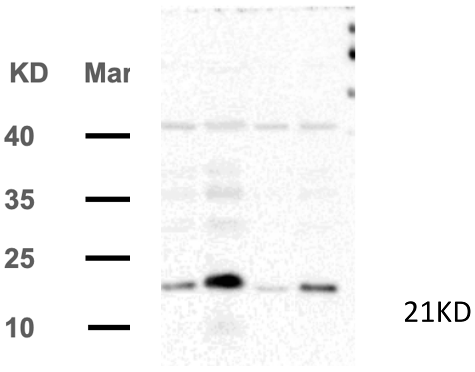

Fig 2 L Bim

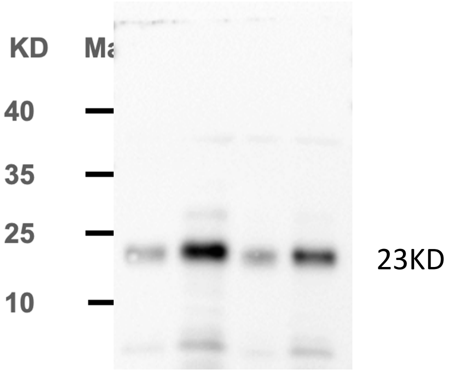

Fig 2 L β-actin

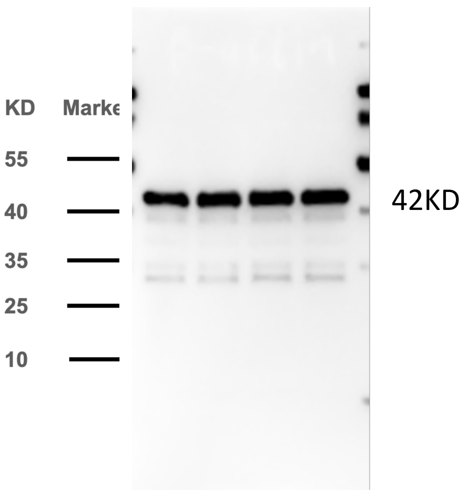

N

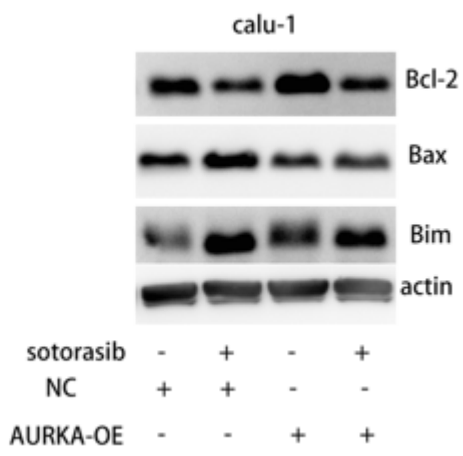

Fig 2 N Bcl-2

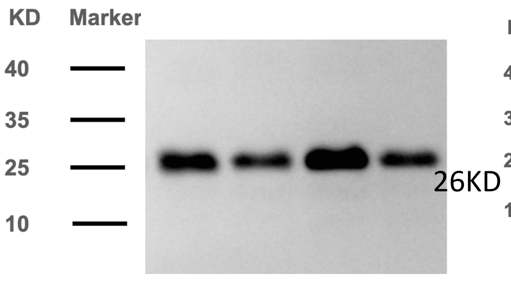

Fig 2 N Bax

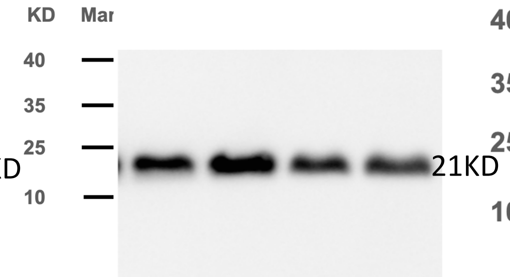

Fig 2 N Bim

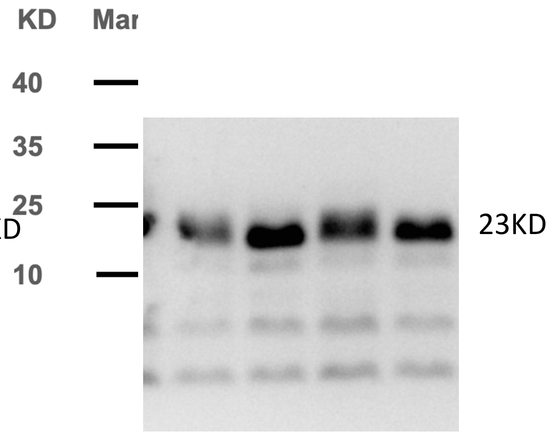

Fig 2 N β-actin

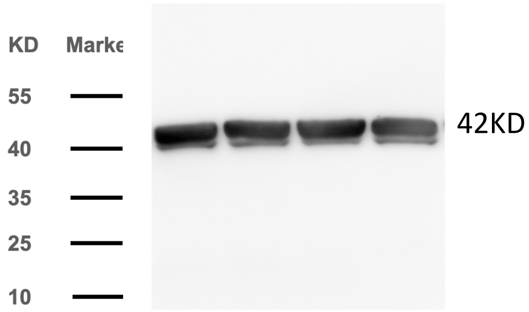

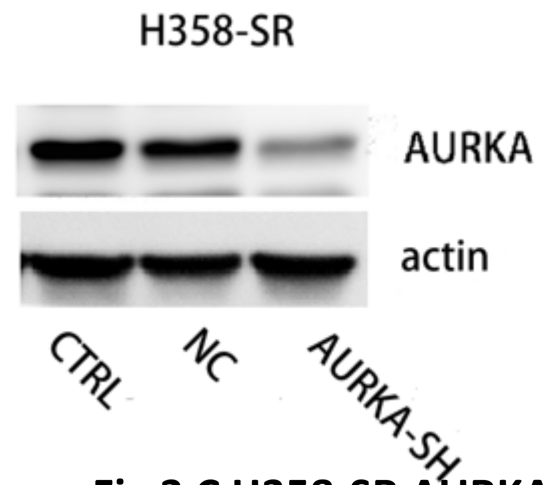

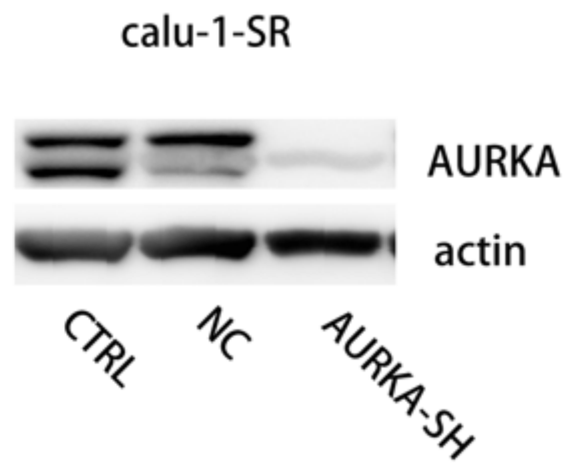

Fig 3 C H358-SR AURKA

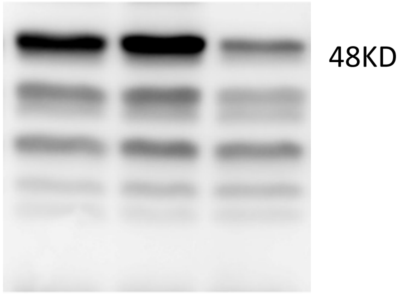

Fig 3 C H358-SR β-actin

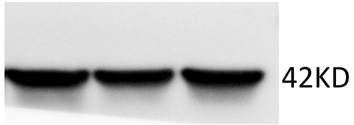

Fig 3 D Calu-SR AURKA

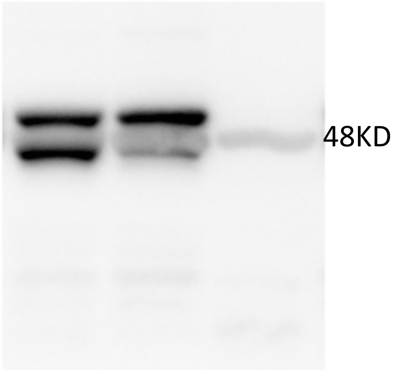

Fig 3 D Calu-SR β-actin

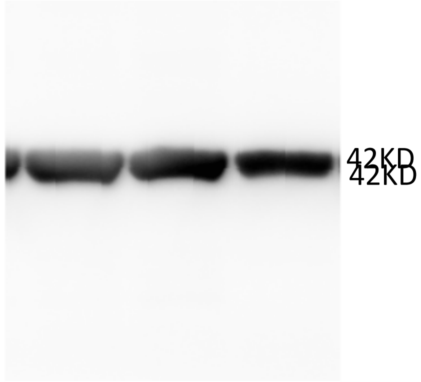

L

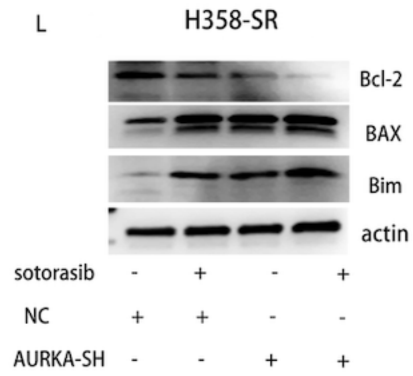

Fig 3 L β-actin

Fig 3 L Bcl-2

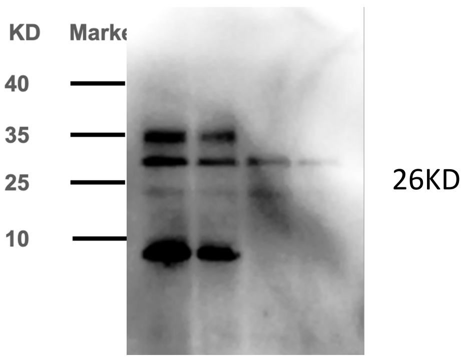

Fig 3 L Bax

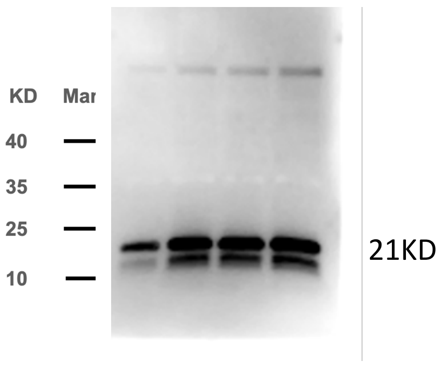

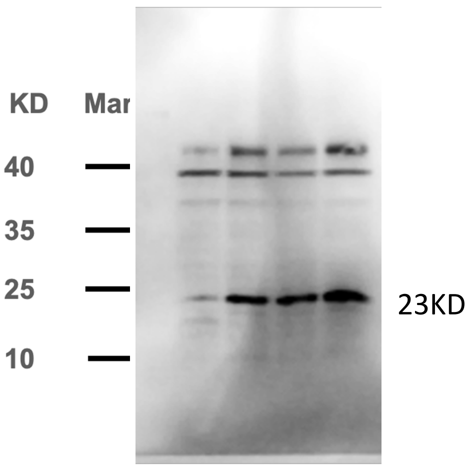

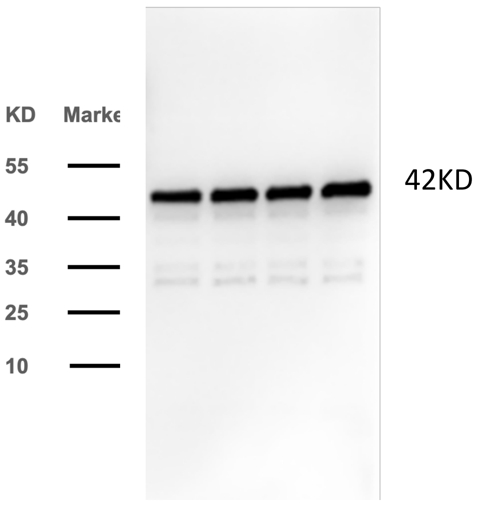

N

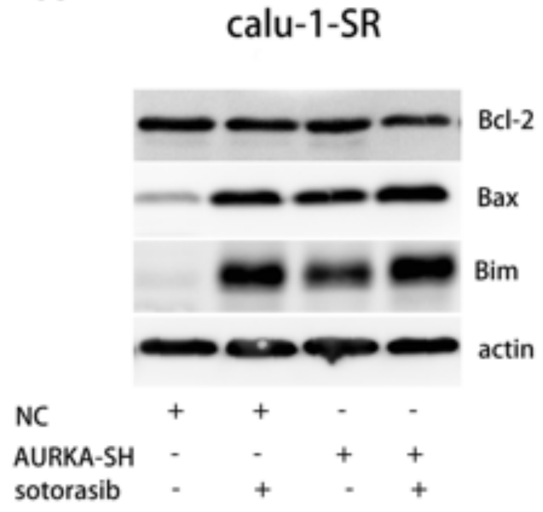

Fig 3 N Bcl-2

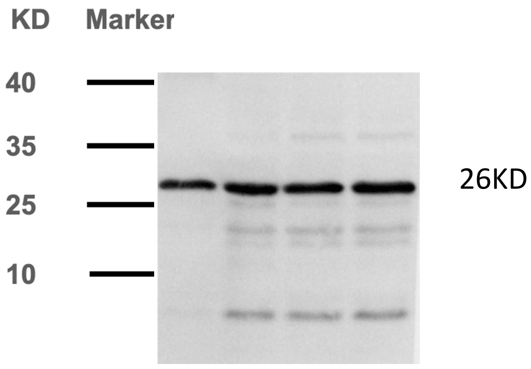

Fig 3 N Bax

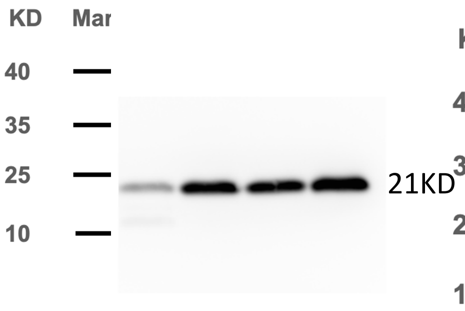

Fig 3 N Bim

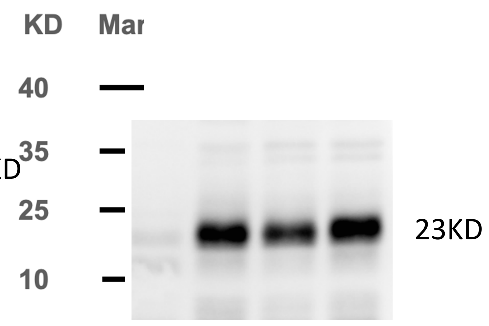

Fig 3 N β-actin

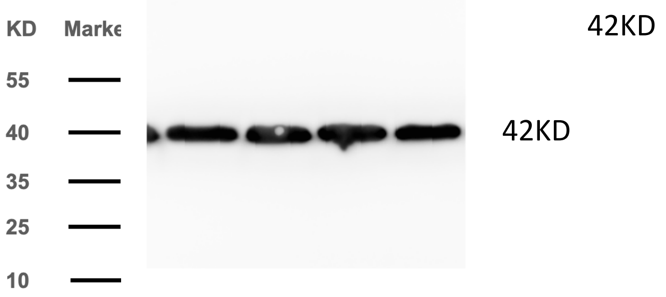

B

C

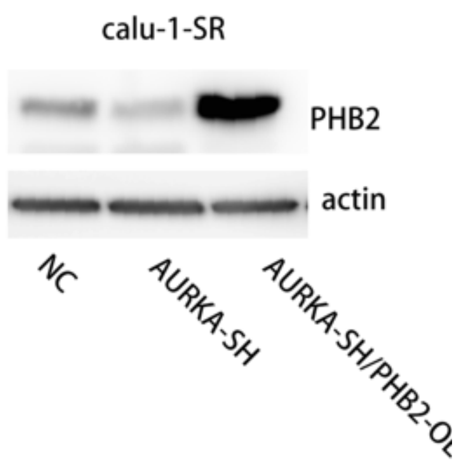

Fig 4 B PHB2

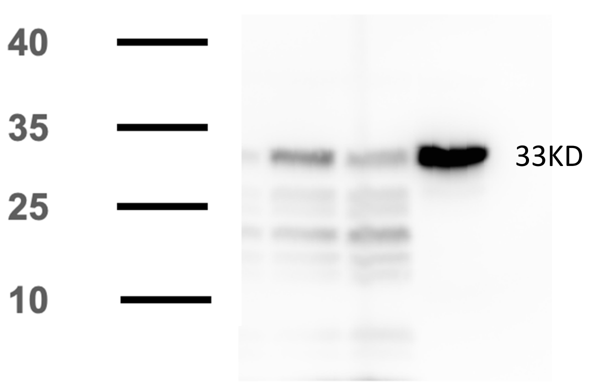

Fig 4B β-actin

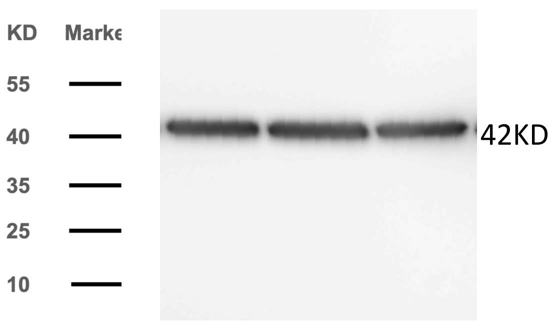

F

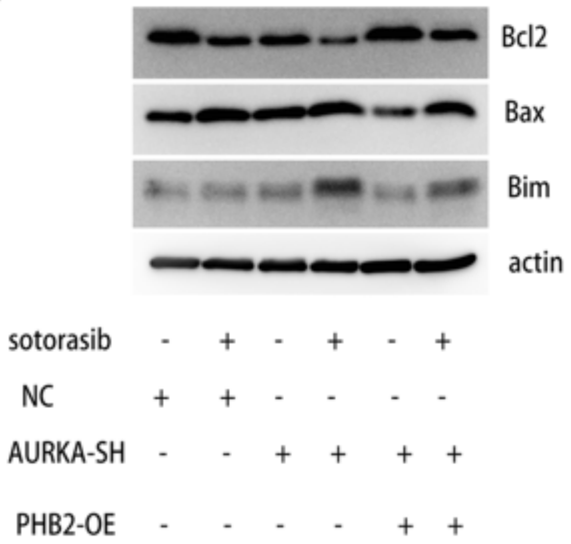

Fig 4 F Bcl-2

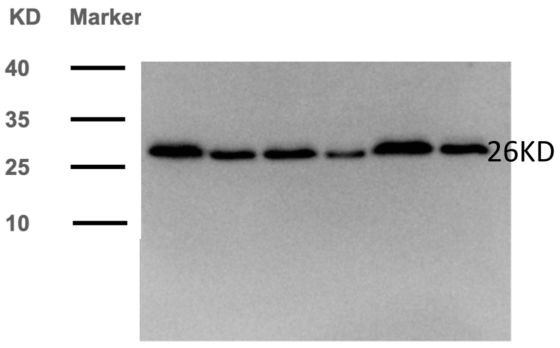

Fig 4F N Bax

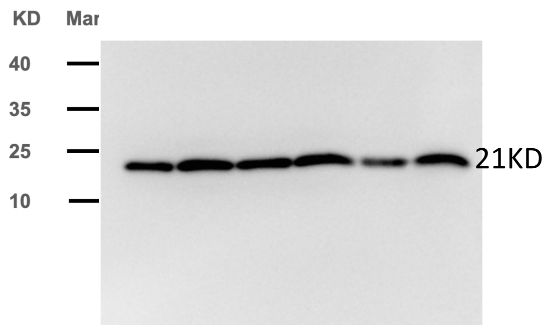

Fig 4F Bim

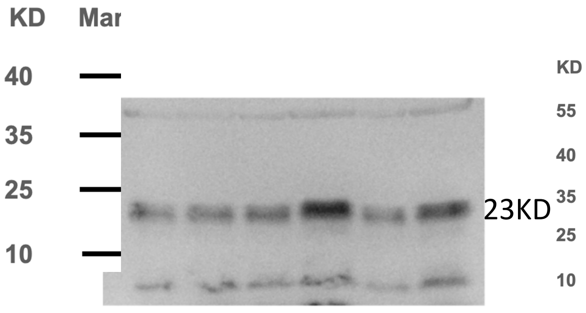

Fig 4F β-actin

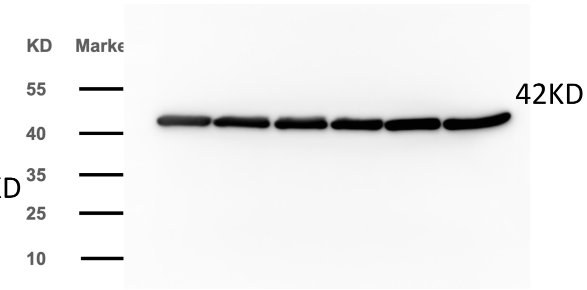

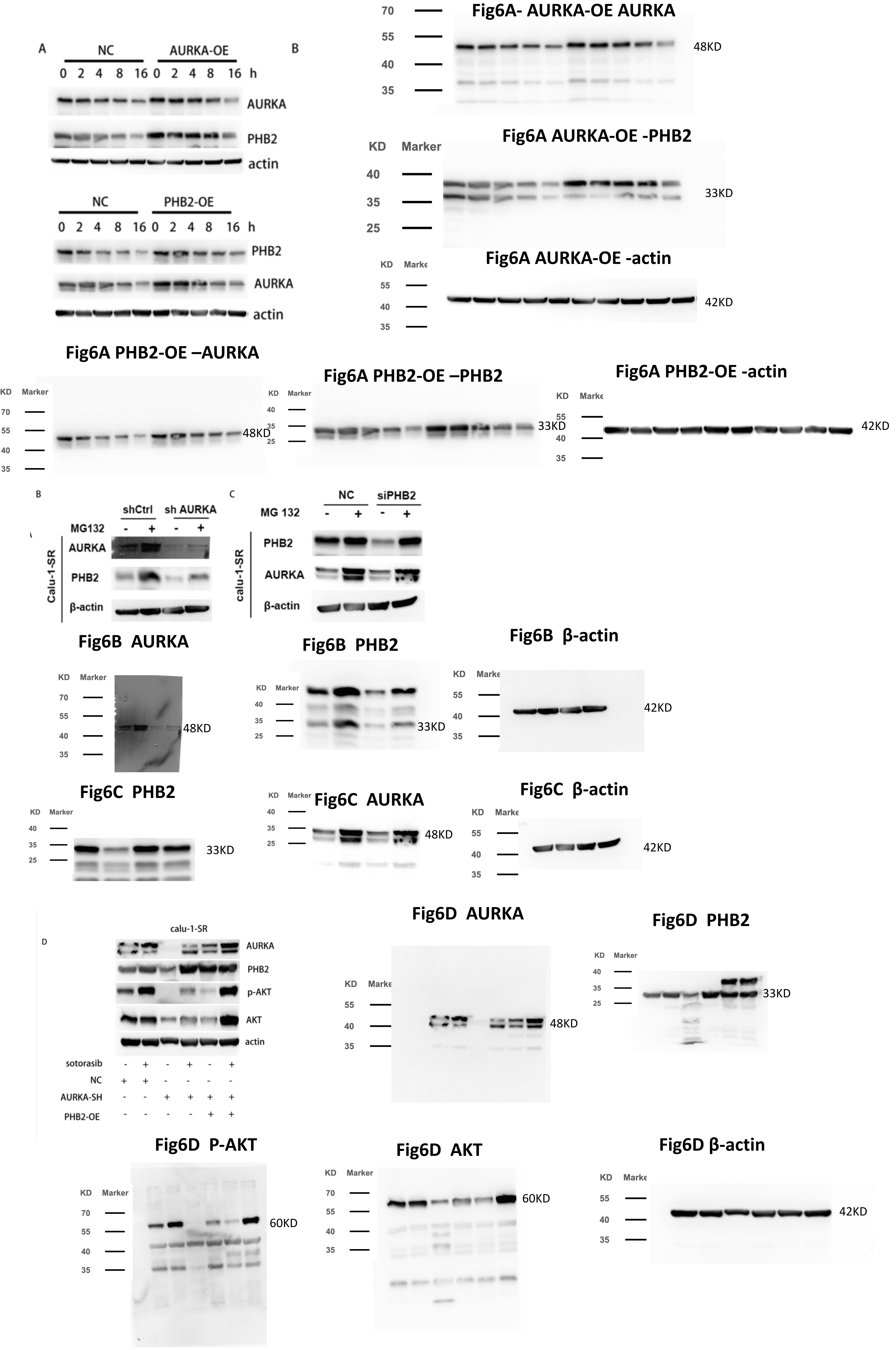
A
NC
AURKA-OE
0 2 4 8 16 0 2 4 8 16 h
AURKA
PHB2
actin
NC
PHB2-OE
0 2 4 8 16 0 2 4 8 16 h
PHB2
AURKA
actin
B
Fig6A- AURKA-OE AURKA
70
55
40
35
48KD
Fig6A AURKA-OE -PHB2
KD Marker
40
35
25
33KD
Fig6A AURKA-OE -actin
KD Marke
55
40
35
42KD
Fig6A PHB2-OE –AURKA
KD Marker
70
55
40
35
48KD
Fig6A PHB2-OE –PHB2
KD Marker
40
35
25
33KD
Fig6A PHB2-OE -actin
KD Marke
55
40
35
42KD
B
shCtrl
sh AURKA
MG132 - + - +
Calu-1-SR
AURKA
PHB2
β-actin
C
NC
siPHB2
MG 132 - + - +
calu-1-SR
PHB2
AURKA
β-actin
Fig6B AURKA
KD Marker
70
55
40
35
48KD
Fig6B PHB2
KD Marker
40
35
25
33KD
Fig6B β-actin
KD Marke
55
40
35
42KD
Fig6C PHB2
KD Marker
40
35
25
33KD
Fig6C AURKA
KD Marker
40
35
25
48KD
Fig6C β-actin
KD Marke
55
40
35
42KD
D
calu-1-SR
AURKA
PHB2
p-AKT
AKT
actin
sotorasib - + - + - +
NC + + - - - -
AURKA-SH - - + + + +
PHB2-OE - - - - + +
Fig6D AURKA
KD Marke
55
40
35
48KD
Fig6D PHB2
KD Marker
40
35
25
33KD
Fig6D P-AKT
KD Marker
70
55
40
35
60KD
Fig6D AKT
KD Marker
70
55
40
35
60KD
Fig6D β-actin
KD Marke
55
40
35
42KD

A

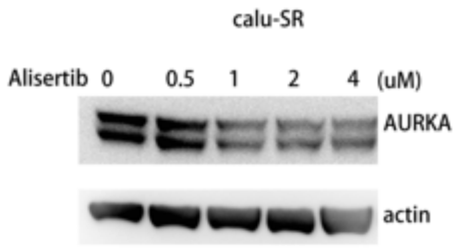

Fig7 A-AURKA

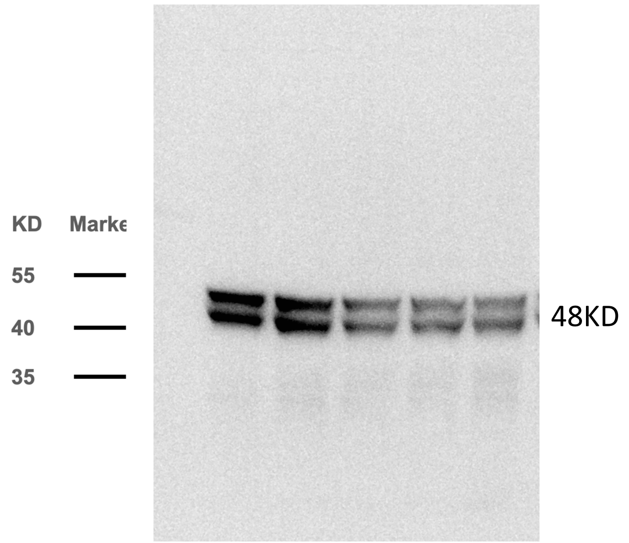

Fig7 A-β-actin

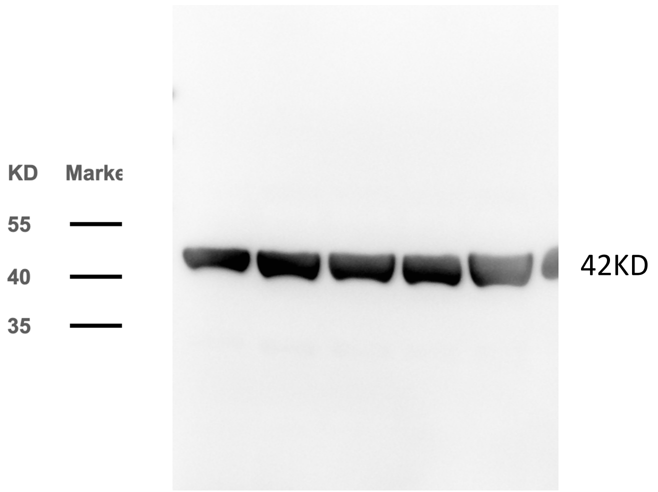

D

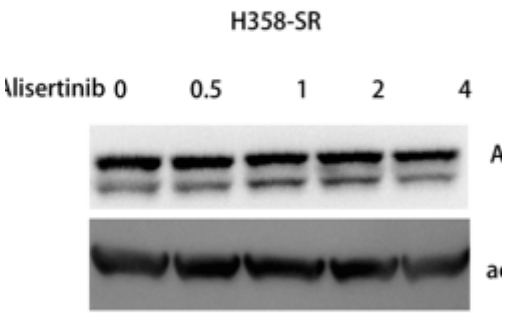

Fig7 D-AURKA

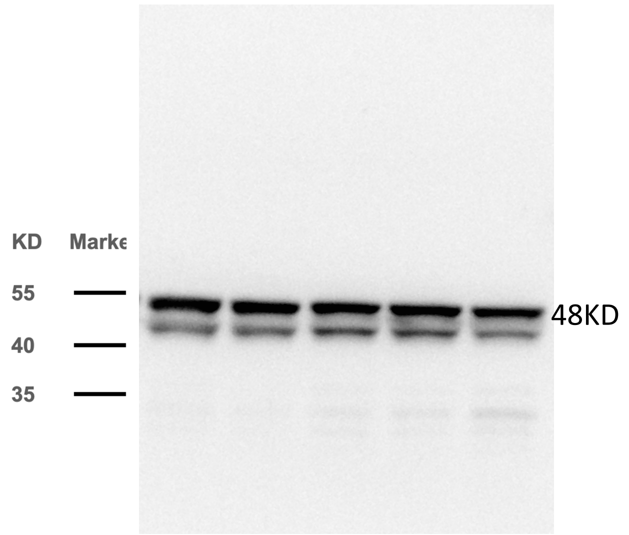

Fig7 D-β-actin

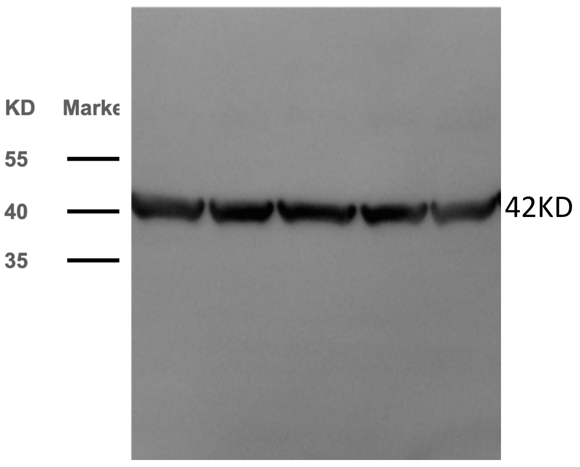

Supplement: Supplementary file 2 — Full and uncropped western blots [file 41420_2026_3080_MOESM2_ESM.pdf]
